# Novel Harziane Diterpenes from Deep-Sea Sediment Fungus *Trichoderma* sp. SCSIOW21 and Their Potential Anti-Inflammatory Effects

**DOI:** 10.3390/md19120689

**Published:** 2021-12-01

**Authors:** Hongxu Li, Xinyi Liu, Xiaofan Li, Zhangli Hu, Liyan Wang

**Affiliations:** 1 Shenzhen Key Laboratory of Marine Bioresource and Eco-Environmental Science, College of Life Sciences and Oceanography, Shenzhen University, Shenzhen 518060, China; lhx@szu.edu.cn (H.L.); 2060251016@email.szu.edu.cn (X.L.); huzl@szu.edu.cn (Z.H.); 2 Key Laboratory of Optoelectronic Engineering, Shenzhen University, Shenzhen 518060, China

**Keywords:** *Trichoderma*, harziane diterpenes, NO inhibition

## Abstract

Five undescribed harziane-type diterpene derivatives, namely harzianol K (**1**), harzianol L (**4**), harzianol M (**5**), harzianol N (**6**), harzianol O (**7**), along with two known compounds, hazianol J (**2**) and harzianol A (**3**) were isolated from the deep-sea sediment-derived fungus *Trichoderma* sp. SCSIOW21. The relative configurations were determined by meticulous spectroscopic methods including 1D, 2D NMR spectroscopy, and HR-ESI-MS. The absolute configurations were established by the ECD curve calculations and the X-ray crystallographic analysis. These compounds (**1**, and **4**–**7**) contributed to increasing the diversity of the caged harziane type diterpenes with highly congested skeleton characteristics. Harzianol J (**2**) exhibited a weak anti-inflammatory effect with 81.8% NO inhibition at 100 µM.

## 1. Introduction

The *Trichoderma* fungus, widely distributed in terrestrial and marine habitats, is a kind of important renewable natural resource with high economic value and application prospects. Among them, the species in the marine environment, together with *Penicillium* and *Aspergillus*, contributed to the discovery of more than half of the new terpenoids from marine fungi [1,2]. However, *Trichoderma* was rarely reported from deep marine ecosystems. During 2013 to 2019, a total of 151 novel compounds were reported from deep marine derived-fungi, of which 41.2% were from *Penicillium*, 28.1% were from *Aspergillus*, while only 1 *Trichoderma* was reported from the deep marine system [1].

Harziane-type diterpenes, containing unique tetracyclic 6-5-4-7 carbon skeleton with 5–6 contiguous stereocenters, are rarely encountered in other organisms. The unprecedented skeleton was initially discovered in 1992 from *Trichoderma harzianum* Rifai [3]. To date, only 44 harziane diterpenes have been reported, almost all of which were discovered solely from *Trichoderma* sp., except for heteroscyphsic acid A from Chinese liverwort *Heteroscyphus coalitus* [4]. These compounds exhibited extensive bioactivities, including anti-bacterial [5,6,7,8,9,10], cytotoxic [8,11,12,13], anti-inflammatory [13,14], anti-HIV [14], phytotoxic [15], algicidal [5,7,16,17], and marine zooplankton toxic activities [6,16] (Table S1 and Figure S1).

During our ongoing investigations on inhibitors from deep-sea fungi [18,19,20,21,22,23] against nitric oxide (NO) production induced by lipopolysaccharide (LPS), *Trichoderma* sp. SCSIOW21, which was isolated from sea sediment at a depth of over 1000 m, was found to be active. The subsequent cultivation of this strain resulted in the isolation of seven harziane diterpenes, including five new compounds. Herein, we report the isolation and identification procedures, as well as the anti-inflammatory, anti-fungal, and anti-bacterial activities of these compounds.

## 2. Results and Discussion

**Figure 1:**
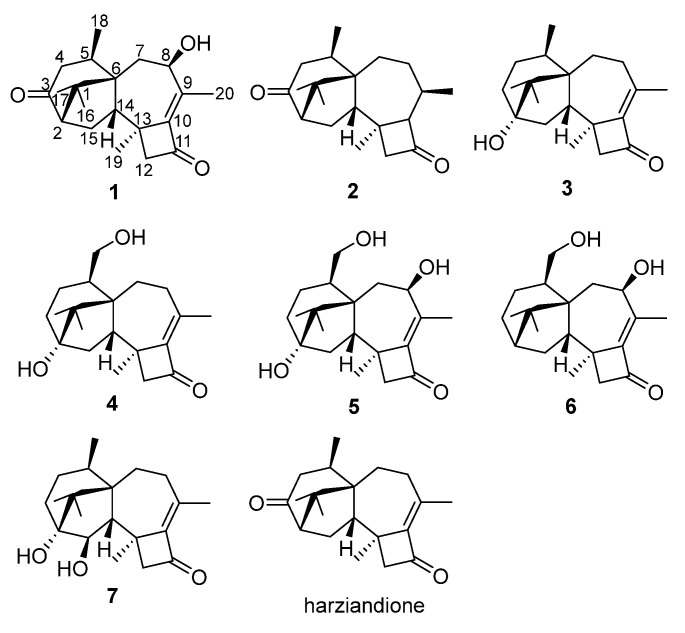
Compounds **1**–**7** and harziandione.

The fungus *Trichoderma* sp. SCSIOW21 was cultured at room temperature under static conditions. The BuOH extraction was fractioned and purified by silica gel, medium pressure ODS column chromatography, and semi-preparative HPLC to obtain seven harziane diterpenes (Figure 1).

**Figure 2:**
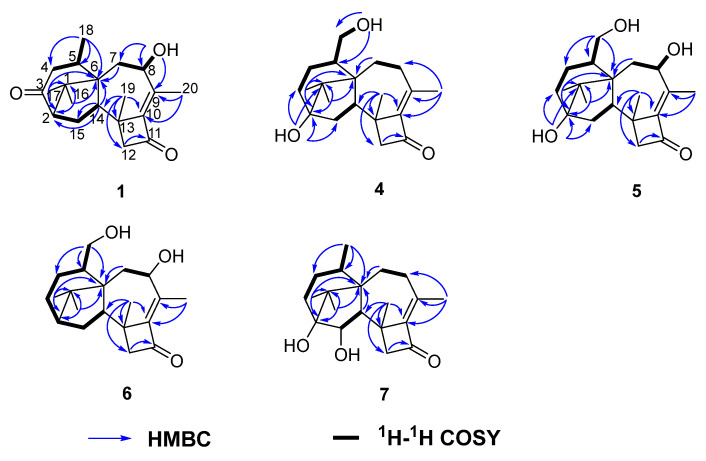
Key 2D NMR spectroscopy correlations of compounds **1** and **4**–**7**.

Compound **1** was isolated as colorless crystal, with molecular formula as C_20_H_28_O_3_ using HRESIMS data. The IR spectrum showed strong absorption bands for two carbonyl groups at 1734 and 1695 cm^−1^, which was consistent with those reported for harziandione [3]. The ^1^H NMR and ^13^C NMR spectroscopy spectra along with HSQC data suggested five methyls, four methylenes, four methines, and seven quaternary carbon atoms (Table 1 and Table 2). The above NMR spectroscopy signal pattern was similar to the prior report for harziandione [3], except for 3 major differences: an additional hydroxy group at δ 5.31, an absent methylene group, and an extra hydroxy group at δ 4.24 compared with harziandione. The up-field shifts of H-8 to δ 4.24 and C-8 to δ 72.4 suggested this group connected to C-8 (Table 1 and Table 2). ^1^H-^1^H COSY correlations between 8-OH and H-8, H-8 and H-7, as well as HMBC correlations from 8-OH to C-8 and C-7 also confirmed the elucidation (Figure 2). This conclusion was further secured by careful analysis of 1D, 2D NMR spectroscopy data, and compound **1** was named as harzianol K, with the molecular framework shown in Figure 2.

**Figure 3:**
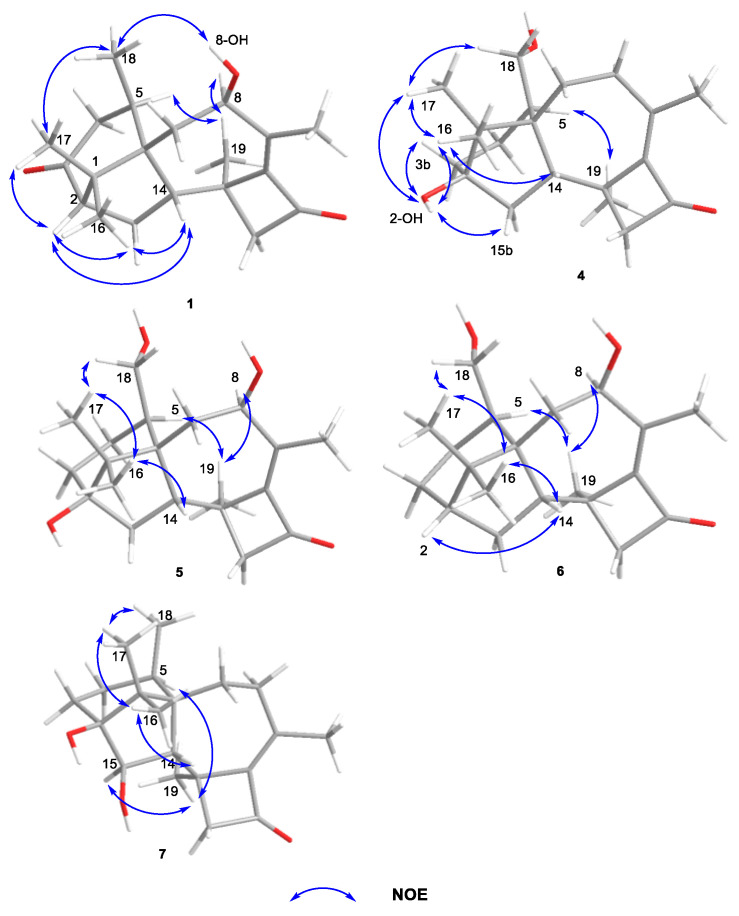
Key NOE correlations of compounds **1** and **4**–**7**.

The relative configuration of **1** was determined by ^1^H-^1^H ROESY spectrum. The ^1^H-^1^H correlations—H-14 and H-2, H-14 and Me-16, H-5 and Me-19, Me-18 and 8-OH—indicated that H-2, H-14, Me-16, Me-17, and Me-18 were located on one side of the molecule, whereas Me-19 and H-5 were located on the opposite side (Figure 3).

**Figure 4:**
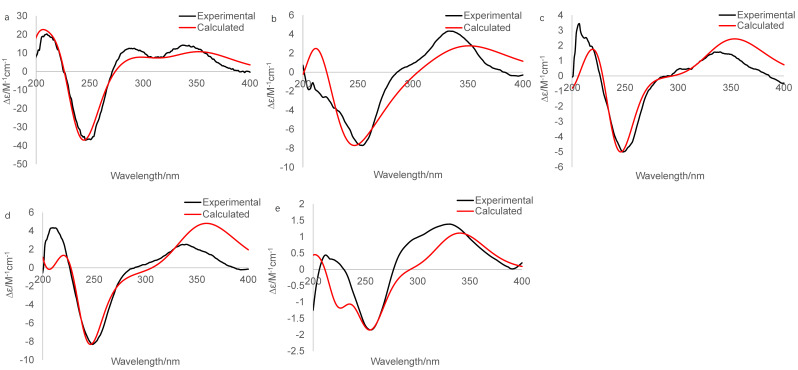
Experimental and calculated (for 2*S*, 5*R*, 6*R*, 8*S*, 13*S*, 14*S*) ECD spectra of **1** (**a**), experimental and calculated (for 2*S*, 5*R*, 6*R*, 13*S*, 14*S*) ECD spectra of **4** (**b**), experimental and calculated (for 2*S*, 5*R*, 6*R*, 8*S*, 13*S*, 14*S*) ECD spectra of **5** (**c**), experimental and calculated (for 2*S*, 5*R*, 6*R*, 8*S*, 13*S*, 14*S*) ECD spectra of **6** (**d**), Experimental and calculated (for 2*S*, 5*R*, 6*R*, 13*S*, 14*S*, 15*S*) ECD spectra of **7** (**e**).

**Figure 5:**
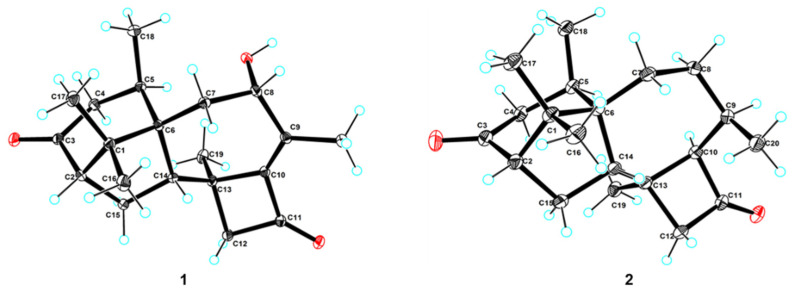
X-ray single-crystallography structures of **1** and **2**. The ellipsoids of non-hydrogen atoms are shown at 30% probability levels for crystal structures.

The experimental CD spectrum of **1** was in accordance with the theoretically calculated ECD curve of the 2*S*, 5*R*, 6*R*, 8*S*, 13*S*, and 14*S* configuration. A total of 3 cotton effects were observed at 245 nm (negative), 292 nm (positive), and 351 nm (positive) (Figure 4a). Eventually, the stereocenters of **1** were determined as 2*S*, 5*R*, 6*R*, 8*S*, 13*S*, and 14*S* unambiguously through analysis of X-ray single-crystallography (Figure 5).

Compounds **2** and **3** were confirmed as known compounds, namely harzianol J [8] and harzianol A [13], by comparing their NMR spectroscopy data with those reported in the literature (Tables S2 and S3) [8]. Nevertheless, the absolute configuration of **2** was not determined previously. Herein we report it as 2*S*, 5*R*, 6*R*, 13*S*, and 14*S* by X-ray diffraction (Figure 5).

Compounds **4**–**7** were all purified as colorless gum or amorphous solids. The molecular formulas of **4**–**7** were established as C_20_H_30_O_3_, C_20_H_30_O_4_, C_20_H_30_O_3_, and C_20_H_30_O_3_ based on HRESIMS data, respectively.

The IR spectrum of **4** showed strong absorption band for carbonyl group at 1716 cm^−1^. The ^1^H and ^13^C NMR spectra of **4** (Table 1 and Table 2) were similar to those of harzianol A (**3**) [13] except for two major differences: the lack of a methyl group and the presence of an extra hydroxy methylene group. The δ_H_ signals at 3.41, 3.28, 4.39 (OH) and the δ_C_ signal at 63.9 suggested that one methyl group was hydroxylated. The ^1^H-^1^H COSY cross-peaks between the hydroxy proton and methylene proton, methylene proton and H-5 (δ_H_ 2.13), along with the HMBC correlations from the hydroxy proton to C-5 (δ_C_ 40.1) and C-18 (δ_C_ 63.9), proved the hydroxy group connected to C-18 unambiguously. The molecular framework of **4** was consequently elucidated as harzianol L (Figure 1 and Figure 2). The relative configuration of **4** was determined by ROESY spectra which showed the same correlation patterns as those of **1** (Figure 3). The absolute configuration of **4** was determined as 2*R*, 5*S*, 6*R*, 13*S*, and 14*S* by comparison of experimental CD spectrum with its calculated ECD data (Figure 4b).

The IR spectrum of **5** showed strong absorption band for carbonyl group at 1732 cm^−1^. The NMR spectroscopy data of **5** was almost consistent with those of **4**, except that a methylene group was missing, whereas an extra oxygenated methine group (δ_H_ 4.21 and δ_C_ 73.5) was detected. The signals suggested that one methylene group was oxygenated (Table 1 and Table 2). ^1^H-^1^H COSY correlations between the hydroxy proton and H-8, between H-8 and H-7, confirmed the connection of the hydroxy group to C-8. The structure was then determined as harzianol M by a detailed analysis of 2D NMR data (Figure 1 and Figure 2). In the ROESY spectra, H-8 showed correlations with Me-19, indicating the β configuration of the 8-hydroxy group (Figure 3). The absolute configurations of **5** were established as 2*R*, 5*S*, 6*R*, 8*S*, 13*S*, and 14*S* based on ECD calculation (Figure 4c).

The IR spectrum of **6** showed a strong absorption band for carbonyl group at 1734 cm^−1^. The NMR spectroscopy spectra of **6** matched well with those of **5**, with just 1 more extra methine group (δ_H_ 2.26 and δ_C_ 51.8) and 1 less oxygenated quaternary carbon signal (Table 1 and Table 2). ^1^H-^1^H COSY correlations between the methine proton and H-3, H-15 suggested the methine group was located at C-3. The molecular framework of **6** was consequently established as harzianol N through a detailed analysis of 2D NMR spectroscopy spectra (Figure 1 and Figure 2). The absolute configurations of **6** were determined as 2*S*, 5*S*, 6*R*, 8*S*, 13*S*, and 14*S* through detailed analysis of ROESY spectra and ECD calculation (Figure 3 and Figure 4d).

The IR spectrum of **7** showed strong absorption band for carbonyl group at 1718 cm^−1^. The ^1^H and ^13^C NMR spectroscopy data of **7** were similar to those reported for harzianol A (**3**) (Table S3) [13], with an extra oxygenated methine group (δ_H_ 3.65 and δ_C_ 73.5) and a disappeared methylene group, indicating the oxygenation of the methylene group (Table 1 and Table 2). The molecular framework was confirmed as harzianol O (Figure 1 and Figure 2) through a detailed analysis of 2D NMR spectroscopy data, including the key COSY correlation between the methine proton and H-14 (δ_H_ 2.07), which suggested the hydroxy group connected to C-15. The ROESY correlations between H-15 and Me-19 suggested the β configuration of the 15-hydroxy group (Figure 3). The absolute configurations of **7** were determined as 2*S*, 5*R*, 6*R*, 13*S*, 14*S*, and 15*R* by ECD calculation.

**Figure 6:**
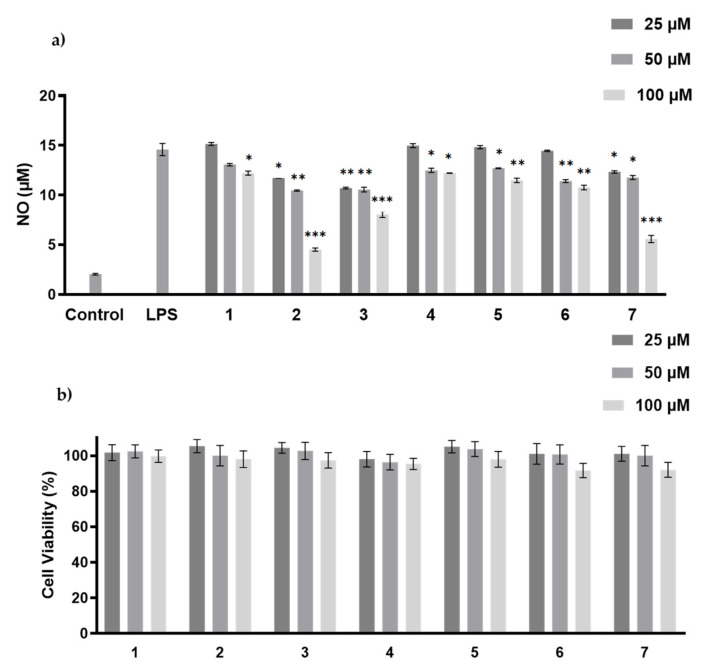
LPS-induced NO production (**a**), and viability (**b**) of RAW 264.7 macrophages by **1**–**7** treatment. The values represent the mean ± SEM of three independent experiments. *, *p* < 0.05; **, *p* < 0.01; ***, *p* < 0.001 vs. control.

The anti-inflammatory activity of compounds **1**–**7** was measured by NO production inhibitory assay [20]. The cytotoxicity of these compounds was tested to avoid false-positive results due to cell death, and none of them showed cytotoxicity at the concentrations of 25–100 µM (Figure 6). Hazianol J (**2**), harzianol A (**3**) and harzianol O (**7**) exhibited the strongest NO production inhibitory activity at 100 µM with inhibitory rates at 81.8%, 46.8%, and 50.5%, respectively. The IC_50_ of Hazianol J (**2**) was 66.7 µM, while harzianol L (**4**) and harzianol K (**1**) only showed weak inhibition at the highest concentration of 100 µM (Figure 6). Compounds without “top” hydroxy groups at C-8 and C-18 (**2**,**3**, and **7**) exhibited higher NO production inhibitory activities compared to the compounds with more hydroxy groups (**1**, **4**, **5**, and **6)**. These hydroxy groups may reduce the membrane permeability and reduced the activities.

All of the compounds were examined for their activities against plant pathogenic fungi (*Helminthosporium maydis*, *Gibberella sanbinetti*, *Botrytis cinerea* Pers, *Fusarium oxysporum* *f*. sp*. cucumerinum*, *Penicillium digitatum*). None of the compounds exhibited obvious activities at the test concentration of 100 μg/mL. Since fungi from *Trichoderma* sp. are widely used as bio-control agents, many harziane diterpenes were investigated against plant pathogenic fungi [3,9,10,16,24]. However, the results were controversial. Although harziandione and isoharziandione, the structure of which was latterly revised as harziandione [10], were mentioned as antifungal agents, the activities of the pure compounds were not clarified in the original literature [3,24]. Harzianone was found to be inactive against *Colletotrichum lagenarium* and *Fusarium oxysporum* at 30 µg/disk using a disk diffusion assay [10]. Deoxytrichodermaerin and harzianol A were not active against *Botrytis cinerea*, *Fusarium oxysporum*, *Glomerella cingulata*, and *Phomopsis asparagi* at 40 µg/disk [16]. Harzianone E was not active against *Candida albicans* by traditional broth dilution assay [9]. According to the previous studies and our results, harziane diterpenes did not show anti-fungal activity.

## 3. Materials and Methods

### 3.1. General Experimental Procedures

The NMR spectroscopy spectra were obtained on the Bruker ASCEND 600 MHz NMR spectrometer equipped with CryoProbe (Bruker Biospin GmbH, Rheinstetten, Germany). Optical rotations were recorded on an Anton Paar MCP-100 polarimeter (Anton Paar GmbH, Austria), with MeOH as solvent. UV spectra were recorded on a UV-1800 spectrometer (Shimadzu Co., Kyoto, Japan). IR spectra were measured on the Nicolet 6700 spectrometer (Thermo, Madison, WI, USA). CD spectra were measured on a J-815 spectropolarimeter (Jasco Co., Japan). Crystallographic data was collected on an XtaLAB Pro: Kappa single four-circle diffractometer using Cu Kα radiation (Rigaku Co., Tokyo, Japan). HRESIMS spectra data were recorded on a MaXis quadrupole-time-of-flight mass spectrometer (Bruker Biospin GmbH, Rheinstetten, Germany). Normal and reverse phase column chromatography (C. C.) was performed using silica gel (200–300 mesh, Qingdao Haiyang Chemical, Qingdao, China) and ODS (YMC Co., Ltd., Kyoto, Japan), respectively. Normal and reverse phase thin-layer chromatography (TLC) was conducted using silica gel 60 F_254_ and RP-18 F_254_ (Merck Millipore Co., Darmstadt, Germany). HPLC was performed using Shimadzu LC-16P system (Shimadzu Co., Kyoto, Japan) with YMC-ODS-A C_18_ Column (20 × 250 mm, 5 µm) for separation. Analytical and HPLC grade reagents (Macklin Co., Shanghai, China) were used for isolation procedures.

### 3.2. Fungal Strain and Fermentation

The fungal strain, which was isolated from the South China deep-sea sediment sample (2134 m depth), was identified as *Trichoderma* sp. SCSIOW21 by ITS sequencing and morphology analysis. Its sequence data was deposited at GenBank (accession number: KC569351.1) and the strain was deposited at the Laboratory of Microbial Natural Products, Shenzhen University, China. The fungal strain was activated on potato dextrose agar dishes containing 3% sea salt at 28 °C for 3 days and cultured in modified rice broth (rice 50.0 g sprayed with 3% sea salt water 60.0 mL for each 500 mL flask) statically at room temperature for 30 days.

### 3.3. Extraction and Isolation

A total of 100 mL of water saturated BuOH were added in each of the Erlenmeyer flasks which contained fermentation broth. The BuOH extract was collected after 12 h and evaporated under vacuum. The extraction was repeated three times and the total yield was 12.9 g.

The BuOH extract was subjected to a silica gel chromatography with a gradient of CH_2_Cl_2_-MeOH-Water (100:0:0, 50:1:0, 20:1:0, 10:1:0, 5:1:0.1, 3:1:0.1, 1:1:0.1, and 0:0:100, *v/v/v*, 2.0 L each) to give 8 fractions (A–H). Fraction B and C were combined and subjected to a medium pressure ODS column with a gradient of MeOH-Water (5:5, 6:4, 7:3, 8:2, and 9:1) to give 5 subfractions. Subfraction 2 was separated by a semi-preparative HPLC column (Acetonitrile (ACN)-Water, 40:60) to give compound **1** (*t*_R_ 52.1 min, 9.0 mg). Subfraction 3 was purified by a semi-preparative HPLC (ACN-Water, 47:53) to give compounds **2** (*t*_R_ 49.2 min, 3.0 mg), **7** (*t*_R_ 32.8 min, 0.8 mg), and **6** (*t*_R_ 34.1 min, 0.8 mg). Subfraction 5 was separated by a semi-preparative HPLC (ACN-Water, 70:30) to give compound **3** (*t*_R_ 15.9 min, 1.0 mg). Fraction D was subjected to a medium pressure liquid chromatography YMC-ODS-A C_18_ Column with a gradient of MeOH-Water (1:9, 2:8, 3:7, 4:6, 5:5, 6:4, 7:3, 8:2, and 9:1) to give 14 subfractions. Subfraction 7 was purified by a semi-preparative HPLC (ACN-Water, 18:82) to give compound **5** (*t*_R_ 29.6 min, 1.6 mg). Subfraction 9 was purified by a semi-preparative HPLC (ACN-Water, 23:77) to give compound **4** (*t*_R_ 41.5 min, 1.0 mg).

### 3.4. Spectral Data of the Compounds

*Harzianol K* (**1**): colorless crystal; [*α*]^25^_D_ +64.1 (*c* 0.36, MeOH); UV (MeOH) *λ*_max_ (log *ε*) 252 (3.77) nm; ECD (0.12 mg/mL, MeOH) *λ*_max_ (∆*ε*) 245 (−36.7), 292 (+7.4), 351 (+10.6) nm; IR (KBr) *v*_max_ 3402 (s), 2927 (m), 1734 (s), 1695 (s), 1190 (m), 1043 (m) cm^−1^; ^1^H NMR and ^13^C NMR spectroscopy data (DMSO-*d_6_*, 600 and 150 MHz), see Table 1 and Table 2; HREIMS *m/z*: 317.2115 [M + H]^+^ (calcd for C_20_H_29_O_3_, 317.2117).

*Harzianol L* (**4**): colorless gum; [*α*]^25^_D_ +15.3 (*c* 0.28, MeOH); UV (MeOH) *λ*_max_ (log *ε*) 256 (3.89) nm; ECD (0.14 mg/mL, MeOH) *λ*_max_ (∆*ε*) 247 (−36.7), 349 (+2.7) nm; IR (KBr) *v*_max_ 3371(s), 2922 (m), 1716 (s), 1653 (m), 1149 (m), 1056 (m) cm^−1^; ^1^H NMR and ^13^C NMR spectroscopy data (DMSO-*d_6_*, 600 and 150 MHz), see Table 1 and Table 2; HREIMS *m/z*: 319.2278 [M + H]^+^ (calcd for C_20_H_31_O_3_, 319.2273).

*HarzianolM* (**5**): colorless gum; [*α*]^25^_D_ +14.1 (*c* 0.15, MeOH); UV (MeOH) *λ*_max_ (log *ε*) 251 (4.07) nm; ECD (0.15 mg/mL, MeOH) *λ*_max_ (∆*ε*) 245 (−8.2), 358 (+4.8) nm; IR (KBr) *v*_max_ 3360 (s), 2922 (m), 1732 (s), 1668 (m), 1122 (m), 1024 (m) cm^−1^; ^1^H NMR and ^13^C NMR spectroscopy data (DMSO-*d_6_*, 600 and 150 MHz), see Table 1 and Table 2; HREIMS *m/z*: 335.2229 [M + H]^+^ (calcd for C_20_H_31_O_4_, 335.2222).

*Harzianol N* (**6**): amorphous solid; [*α*]^25^_D_ +10.1 (*c* 0.18, MeOH); UV (MeOH) *λ*_max_ (log *ε*) 252 (4.19) nm; ECD (0.18 mg/mL, MeOH) *λ*_max_ (∆*ε*) 220 (+1.7), 245 (−4.9), 353 (+2.4) nm; IR (KBr): *v*_max_ 3379 (s), 2924 (m), 1734 (s), 1647 (m), 1153 (m), 1049 (m) cm^−1^; ^1^H NMR and ^13^C NMR spectroscopy data (DMSO-*d_6_*, 600 and 150 MHz), see Table 1 and Table 2; HREIMS *m/z*: 341.2089 [M + Na]^+^ (calcd for C_20_H_30_NaO_3_, 341.2093).

*HarzianolO* (**7**): amorphous solid; [*α*]^25^_D_ +12.0 (*c* 0.14, MeOH); UV (MeOH) *λ*_max_ (log *ε*) 256 (4.11) nm; ECD (0.14 mg/mL, MeOH) *λ*_max_ (∆*ε*) 255 (−1.8), 340 (+1.1) nm; IR (KBr) *v*_max_ 3360 (s), 2922 (m), 1718 (s), 1660 (m), 1147 (m), 1058 (m) cm^−1^; ^1^H NMR and ^13^C NMR spectroscopy data (DMSO-*d_6_*, 600 and 150 MHz), see Table 1 and Table 2; HREIMS *m/z*: 319.2269 [M + H]^+^ (calcd for C_20_H_31_O_3_, 319.2273).

### 3.5. X-ray Crystal Analysis of Compounds ***1*** and ***2***

The crystals of compounds **1** and **2** were obtained from concentrated MeOH solutions and 1 suitable crystal for each compound was selected. The crystals were scanned using Cu Kα radiation (λ = 1.54184 Å) on the XtaLAB AFC12 (RINC) Kappa single diffraction instrument, the structures of which were solved by the Olex2 software, the SHELXT [25], and the SHELXL [26] package with the parameters corrected by the least-squares minimization method.

The single-crystal data has been submitted to the Cambridge Crystallographic Data Centre database, with CCDC 2093540 for **1** and CCDC 2093541 for **2.** The data can be downloaded for free from the website http://www.ccdc.cam.ac.uk/ (accessed on 7 November 2021).

*X-ray crystal data of***1**: C_20_H_28_O_3_ (*M* = 316.42 g/mol): monoclinic, space group *P*2_1_ (no. 4), a = 8.73030 (10) Å, b = 11.43810 (10) Å, c = 8.99520 (10) Å, β = 110.2970 (10)°, V = 842.468 (16) Å^3^, Z = 2, T = 100.01 (10) K, μ (Cu Kα) = 0.648 mm^−1^, Dcalc = 1.247 g/cm^3^, 8392 reflections measured (10.486° ≤ 2ϴ ≤ 148.666°), 3306 unique (R_int_ = 0.0193, R_sigma_ = 0.0222) which were used in all calculations. The final R_1_ was 0.0273 [I > 2σ(I)] and wR_2_ was 0.0705 (all data), Flack parameter 0.04 (5).

*X-ray crystal data of***2**: C_40_H_60_O_4_ (*M* = 604.88 g/mol): monoclinic, space group P2_1_ (no. 4), a = 7.84120 (10) Å, b = 9.31180 (10) Å, c = 23.1108 (2) Å, β = 93.9960 (10)°, V = 1683.35 (3) Å^3^, Z = 2, T = 100.01(10) K, μ(Cu Kα) = 0.576 mm^−1^, Dcalc = 1.193 g/cm^3^, 19,167 reflections measured (7.67° ≤ 2 ϴ ≤ 148.826°), 6578 unique (R_int_ = 0.0295, R_sigma_ = 0.0304) which were used in all calculations. The final R_1_ was 0.0343 [I > 2σ(I)] and wR_2_ was 0.0890 (all data), Flack parameter 0.03 (9).

### 3.6. ECD Computational Methods

The conformations of compounds **1** and **4**–**7** were searched by Marvin Sketch software (optimization limit = normal, diversity limit = 0.1) ignoring the rotation of methyl and hydroxy groups. Geometric optimization of the molecules in MeOH (Figures S49–S53) was carried out at 6-31G (d, p) level using DFT/B3LYP through Gaussian 09 software [27], within the 3 kcal/mol energy threshold from the global minimum [28]. The ECD curve was simulated based on TD-DFT calculations and drawn with sigma = 0.3 by SpecDis software (version 1.71, Berlin, Germany). The calculated data was also produced by Boltzmann’s weighting and magnetization based on experimental values.

### 3.7. MTT and NO Production Inhibitory Assay

The cytotoxicity and NO production inhibitory activity were examined using RAW 264.7 macrophages, and the detailed methods were reported previously [20].

### 3.8. Anti-Fungal Activities

The anti-fungal activities were tested on a 96-well plate by mycelial growth inhibitory assay [29], using actidione as the positive control. Five plant pathogenic fungal species (*Helminthosporium maydis,* *Gibberella sanbinetti,* *Botrytis cinerea* Pers*,* *Fusarium Oxysporum* f. sp*. cucumerinum,* *Penicillium digitatum*) were donated by CAS Key Laboratory of Tropical Marine Bio-resources and Ecology, Chinese Academy of Sciences.

## 4. Conclusions

Herein, we reported the isolation, structure elucidation, and biological activities of seven harziane diterpenes, including five new compounds from a deep-sea derived fungus, *Trichoderma* sp. SCSIOW21. The stereo configurations of the new compounds, harzianol K (**1**), harzianol L (**4**), harzianol M (**5**), harzianol N (**6**), and harzianol O (**7**) were characterized by ECD calculations. Hazianol K (**1**) and harzianol J (**2**) were unambiguously determined by X-ray single crystallographic analysis. Hazianol J (**2**), harzianol A (**3**), and harzianol O (**7**) exhibited weak NO production inhibitory activity. All of the compounds did not show any anti-fungal activities.

## Figures and Tables

**Table 1:** ^1^H NMR spectroscopy (600 MHz) ^a^ of compounds **1**, **4**–**7**.

	1	4	5	6	7
No.	δ_H_ (*J* in Hz)	δ_H_ (*J* in Hz)	δ_H_ (*J* in Hz)	δ_H_ (*J* in Hz)	δ_H_ (*J* in Hz)
1					
2	2.06, d (8.0)			2.26, dd (11.0, 8.0)	
2-OH		4.17, s	4.14, s		
3α		1.81, m ^b^	1.77, m	1.78, m	1.80, m
3β		1.32, dd (12.0, 7.0)	1.30, dd (12.0, 7.0)	1.23, m	1.31, m ^b^
4α	2.92, dd (17.0, 11.0)	1.80, m	1.85, m	1.89, m	1.85, m
4β	1.84, d (17.0)	1.64, d (12.0)	1.60, m	1.48, dd (14.0, 6.0)	1.34, m
5	3.38, m	2.13, t (8.0)	2.71, t (8.0)	2.75, t (8.0)	2.32, m ^b^
6					
7α	2.16, dd (15.0, 5.0)	1.76, m ^b^	2.11, dd (15.0, 5.0)	2.14, dd (15.0, 5.0)	2.35, m ^b^
7β	1.36, dd (15.0, 2.0)	1.28, m	1.36, dd (15.0, 2.0)	1.30, dd (15.0, 2.0)	1.90, ddd (13.0, 7.0, 2.0)
8α	4.24, d (5.0, 2.0)	2.52, m	4.21, dd (5.0, 2.0)	4.22, dd (5.0, 2.0)	1.98, dd (13.0, 7.0)
8β		1.88, ddd (16.0, 6.0, 2.0)			1.29, m ^b^
8-OH	5.31, brs		5.45, brs		
9					
10					
11					
12α	2.77, d (16.0)	2.60, d (16.0)	2.65, d (16.0)	2.71, d (16.0)	2.98, d (16.0)
12β	2.33, d (16.0)	2.26, d (16.0)	2.29, d (16.0)	2.33, d (16.0)	2.34, d (16.0)
13					
14	2.57, dd (11.0, 9.0)	2.21, dd (12.0, 9.0)	2.33, dd (12.0, 9.0)	1.56, m	2.07, d (6.0)
15α	1.98, m	1.58, dd (13.0.0, 9.0)	1.65, m	1.84, m	3.65, d (6.0)
15β	1.49, dd (14.0, 9.0)	1.69, dd (13.0,12.0)	1.57, m	1.35, m	
16	0.93, s	0.81, s	0.82, s	0.80, s	0.84, s
17	0.91, s	0.66, s	0.70, s	0.84, s	0.89, s
18α	1.18, d (7.0)	3.41, m	3.85, d (10.0)	3.89, d (10.0)	0.99, d (7.0)
18β		3.28, m	3.23, m	3.26, m	
18-OH		4.39, t (6.0)			
19	1.53, s	1.39, s	1.46, s	1.51, s	1.43, s
20	2.04, s	2.01, s	2.03, s	2.03, s	2.02, s

^a^ Recorded in DMSO-*d_6_*; ^b^ overlapped signals.

**Table 2:** ^13^C NMR spectroscopy (150 MHz) ^a^ data of Compounds **1**, **4**–**7**.

	1	4	5	6	7
No.	δ_C_, Type	δ_C_, Type	δ_C_, Type	δ_C_, Type	δ_C_, Type
1	50.5, C	48.5, C	49.2, C	51.1, C	48.2, C
2	58.9, CH	77.9, C	77.4, C	51.8, CH	75.9, C
3	213.6, C	33.2, CH_2_	33.5, CH_2_	25.5, CH_2_	30.4, CH_2_
4	43.2, CH_2_	22.6, CH_2_	23.9, CH_2_	22.0, CH_2_	25.2, CH_2_
5	31.4, CH	40.1, CH	41.7, CH	42.4, CH	27.5, CH
6	51.7, C	52.7, C	53.1, C	45.7, C	50.7, C
7	33.0, CH_2_	30.2, CH_2_	34.3, CH_2_	33.7, CH_2_	29.3, CH_2_
8	72.4, CH	29.3, CH_2_	73.5, CH	73.1, CH	31.5, CH_2_
9	144.4, C	145.5, C	143.0, C	143.1, C	145.8, C
10	150.4, C	149.7, C	150.0, C	150.6, C	149.6, C
11	199.6, C	198.2, C	200.0, C	200.1, C	198.2, C
12	58.8, CH_2_	59.2, CH_2_	58.9, CH_2_	58.9, CH_2_	59.1, CH_2_
13	40.1, C	40.0, C	40.7, C	40.9, C	40.0, C
14	52.2, CH_2_	50.6, CH	50.5, CH	42.4, CH	60.1, CH
15	26.1, CH	35.6, CH_2_	35.7, CH_2_	27.1, CH_2_	73.5, CH
16	25.6, CH_3_	19.7, CH_3_	20.1, CH_3_	25.8, CH_3_	20.5, CH_3_
17	23.4, CH_3_	18.9, CH_3_	19.0, CH_3_	21.9, CH_3_	19.7, CH_3_
18	22.8, CH_3_	63.9, CH_2_	65.6, CH_2_	65.9, CH_2_	19.9, CH_3_
19	20.2, CH_3_	21.6, CH_3_	21.0, CH_3_	21.0, CH_3_	22.3, CH_3_
20	20.1, CH_3_	22.0, CH_3_	20.2, CH_3_	20.2, CH_3_	21.9, CH_3_

^a^ Recorded in DMSO-*d_6._*

## Data Availability

Not applicable.

## Supplementary Materials

The following are available online at https://www.mdpi.com/article/10.3390/md19120689/s1, including detailed 1D and 2D NMR data, ECD calculations, HRESIMS spectra for compounds **1**–**7**, as well as a brief summary of reported literatures about harziane type diterpenes from 1992 to 2021.
