# Supplementary material for: Novel Harziane Diterpenes from Deep-Sea Sediment Fungus Trichoderma sp. SCSIOW21 and Their Potential Anti-Inflammatory Effects
Source: Mar Drugs. 2021 Dec 1;19(12):689. doi: 10.3390/md19120689 (PMC8705903; doi:10.3390/md19120689)
Supplement: Supplementary file 1 [file marinedrugs-19-00689-s001.zip › marinedrugs-1476180-supplementary.pdf]

Supporting Information

Novel Harziane Diterpenes from Deep-Sea Sediment Fungus *Trichoderma* sp. SCSIOW21 and their Potential Anti-Inflammatory Effects

Hongxu Li [1,2], Xinyi Liu[1], Xiaofan Li [1,*],Zhangli Hu[1,2]and Liyan Wang [1,*]

[1] Shenzhen Key Laboratory of Marine Bioresource and Eco-environmental science, College of Life Sciences and Oceanography, Shenzhen University, Shenzhen 518060, China; lhx@szu.edu.cn (H. L.); (X. L.); (X. L.); (Z. H.); (L. W.)

[2] Key Laboratory of Optoelectronic Engineering, Shenzhen University, Shenzhen 518060, China

***** Correspondence: lwang@szu.edu.cn (L. W.); lixf@szu.edu.cn (X. L.) Tel.: +86-755-2601-2653

List of Supporting Information

Table S1. Reported literatures about harziane type diterpenes from 1992 to 2021.

Table S2. ^{1}H NMR (600 MHz) and ^{13}C NMR (150 MHz) data for compound **2** in DMSO-d_6.

Table S3. ^{1}H NMR (600 MHz) and ^{13}C NMR (150 MHz) data for compound **3** in DMSO-d_6.

Table S4. Cartesian coordinates for the lowest-energy conformer of compound **1** in ECD calculation.

Table S5. Cartesian coordinates for the lowest-energy conformer of compound **4** in ECD calculation.

Table S6. Cartesian coordinates for the lowest-energy conformer of compound **5** in ECD calculation.

Table S7. Cartesian coordinates for the lowest-energy conformer of compound **6** in ECD calculation.

Table S8. Cartesian coordinates for the lowest-energy conformer of compound **7** in ECD calculation.

Figure S1. Reported compounds from previous literatures from 1992 to 2021.

Figure S2. ^{1}H NMR (600 MHz, DMSO-d_6) spectrum of compound **1**, at 25°C.

Figure S3. ^{13}C NMR (150 MHz, DMSO-d_6) spectrum of compound **1**, at 25°C.

Figure S4. ^{1}H-^{1}H COSY (DMSO-d_6) spectrum of compound **1**, at 25°C.

Figure S5. HSQC (DMSO-d_6) spectrum of compound **1**, at 25°C.

Figure S6. HMBC (DMSO-d_6) spectrum of compound **1**, at 25°C.

Figure S7. ROESY (DMSO-d_6) spectrum of compound **1**, at 25°C.

Figure S8. HRESIMS spectrum of compound **1**.

Figure S9. ^{1}H NMR (600 MHz, DMSO-d_6) spectrum of compound **2**, at 25°C.

Figure S10. ^{13}C NMR (150 MHz, DMSO-d_6) spectrum of compound **2**, at 25°C.

Figure S11. ^{1}H-^{1}H COSY (DMSO-d_6) spectrum of compound **2**, at 25°C.

Figure S12. HSQC (DMSO-d_6) spectrum of compound **2**, at 25℃.

Figure S13. HMBC (DMSO-d_6) spectrum of compound **2**, at 25℃.

Figure S14. ROESY (DMSO-d_6) spectrum of compound **2**, at 25℃.

Figure S15. ^{1}H NMR (600 MHz, DMSO-d_6) spectrum of compound **3**, at 25℃.

Figure S16. ^{13}C NMR (150 MHz, DMSO-d_6) spectrum of compound **3**, at 25℃.

Figure S17. ^{1}H-^{1}H COSY (DMSO-d_6) spectrum of compound **3**, at 25℃.

Figure S18. HSQC (DMSO-d_6) spectrum of compound **3**, at 25℃.

Figure S19. HMBC (DMSO-d_6) spectrum of compound **3**, at 25℃.

Figure S20. ROESY (DMSO-d_6) spectrum of compound **3**, at 25℃.

Figure S21. ^{1}H NMR (600 MHz, DMSO-d_6) spectrum of compound **4**, at 25℃.

Figure S22. ^{13}C NMR (150 MHz, DMSO-d_6) spectrum of compound **4**, at 25℃.

Figure S23. ^{1}H-^{1}H COSY (DMSO-d_6) spectrum of compound **4**, at 25℃.

Figure S24. HSQC (DMSO-d_6) spectrum of compound **4**, at 25℃.

Figure S25. HMBC (DMSO-d_6) spectrum of compound **4**, at 25℃.

Figure S26. ROESY (DMSO-d_6) spectrum of compound **4**, at 25℃.

Figure S27. HRESIMS spectrum of compound **4**.

Figure S28. ^{1}H NMR (600 MHz, DMSO-d_6) spectrum of compound **5**, at 25℃.

Figure S29. ^{13}C NMR (150 MHz, DMSO-d_6) spectrum of compound **5**, at 25℃.

Figure S30. ^{1}H-^{1}H COSY (DMSO-d_6) spectrum of compound **5**, at 25℃.

Figure S31. HSQC (DMSO-d_6) spectrum of compound **5**, at 25℃.

Figure S32. HMBC (DMSO-d_6) spectrum of compound **5**, at 25℃.

Figure S33. ROESY (DMSO-d_6) spectrum of compound **5**, at 25℃.

Figure S34. HRESIMS spectrum of compound **5**.

Figure S35. ^{1}H NMR (600 MHz, DMSO-d_6) spectrum of compound **6**, at 25℃.

Figure S36. ^{13}C NMR (150 MHz, DMSO-d_6) spectrum of compound **6**, at 25℃.

Figure S37. ^1H-^1H COSY (DMSO-d_6) spectrum of compound **6**, at 25°C.

Figure S38. HSQC (DMSO-d_6) spectrum of compound **6**, at 25°C.

Figure S39. HMBC (DMSO-d_6) spectrum of compound **6**, at 25°C.

Figure S40. ROESY (DMSO-d_6) spectrum of compound **6**, at 25°C.

Figure S41. HRESIMS spectrum of compound **6**.

Figure S42. ^1H NMR (600 MHz, DMSO-d_6) spectrum of compound **7**, at 25°C.

Figure S43. ^{13}C NMR (150 MHz, DMSO-d_6) spectrum of compound **7**, at 25°C.

Figure S44. ^1H-^1H COSY (DMSO-d_6) spectrum of compound **7**, at 25°C.

Figure S45. HSQC (DMSO-d_6) spectrum of compound **7**, at 25°C.

Figure S46. HMBC (DMSO-d_6) spectrum of compound **7**, at 25°C.

Figure S47. ROESY (DMSO-d_6) spectrum of compound **7**, at 25°C.

Figure S48. HRESIMS spectrum of compound **7**.

Figure S49. Comparison of the calculated ECD spectrum of (2*S*, 5*R*, 6*R*, 8*S*, 13*S*, 14*S*)-**1** at the B3LYP/6-311+G(d, p) level with the experimental ECD spectrum of 1 in MeOH, σ = 0.41 eV, shift = +10 nm.

Figure S50. Comparison of the calculated ECD spectrum of (2*S*, 5*R*, 6*R*, 13*S*, 14*S*)-**4** at the B3LYP/6-311+G(d, p) level with the experimental ECD spectrum of 1 in MeOH, σ = 0.49 eV, shift = +10 nm.

Figure S51. Comparison of the calculated ECD spectrum of (2*S*, 5*R*, 6*R*, 8*S*, 13*S*, 14*S*)-**5** at the B3LYP/6-311+G(d, p) level with the experimental ECD spectrum of 1 in MeOH, σ = 0.40 eV, shift = +15 nm.

Figure S52. Comparison of the calculated ECD spectrum of (2*S*, 5*R*, 6*R*, 8*S*, 13*S*, 14*S*)-**6** at the B3LYP/6-311+G(d, p) level with the experimental ECD spectrum of 1 in MeOH, σ = 0.39 eV, shift = +9 nm.

Figure S53. Comparison of the calculated ECD spectrum of (2*S*, 5*R*, 6*R*, 13*S*, 14*S*, 15*S*)-**7** at the B3LYP/6-311+G(d, p) level with the experimental ECD spectrum of 1 in MeOH, σ = 0.34 eV, shift = -2 nm.

Table S1. Reported literatures about harziane type diterpenes from 1992 to 2021.

References	Publish year	Compound name	Sample source	Bioassays
**[1]**	1992	harziandione	*Trichoderma harzianum* Rifai	-
**[2]**	1997	isoharzandione	*Trichoderma viride*	anti-fungal
**[3]**	2012	harzianone	*Trichoderma longibrachiatum*	anti-bacterial; anti-fungal; brine shrimp toxicity;
**[4]**	2013	trichodermaerin	*Trichoderma erinaceum*	cytotoxic
**[5]**	2014	compounds **2**; **3**; **4**	*Trichoderma atroviridae*	anti-bacterial; cytotoxic
**[6]**	2016	(9*R*,10*R*)-dihydro-harzianelactone; hazrianelactone	*Trichoderma* sp. Xy24	cytotoxic
**[7]**	2017	harzianols A–E; harziane acid	microbial transformation	cytotoxicity; anti-inflammatory; anti-HIV
**[8]**	2017	furanharzianones A and B	microbial transformation	cytotoxicity; anti-inflammatory; anti-HIV
**[9]**	2018	3*R*-hydroxy-9*R*,10*R*-dihydroharzianone	*Trichoderma harzianum* X-5	anti- plankton activities
**[10]**	2018	11-hydroxy-9-harzien-3-one	*Trichoderma asperellum* cf44-2	anti-bacterial; anti- plankton activities
**[11]**	2019	3*S*-hydroxyhazianone	*Trichoderma asperellum* A-YMD-9-2	anti- plankton activities
**[12]**	2019	harzianelactones A and B; harzianones A–D; harziane	*Trichoderma harzianum* XS-20090075	phytotoxicity
**[13]**	2020	harzianols F–J	*Trichoderma atroviridae* B7	anti-bacterial; cytotoxicity
**[14]**	2020	harzianone E	*Trichoderma harzianum* XS-20090075	anti-bacterial; anti-fungal; DNA Topo I inhibition; AChE inhibition

**[15]**	2020	heteroscyphsic acid A	*Heteroscyphus coalitus*	anti-fungal
**[16]**	2020	harziandione A	*Trichoderma erinaceum* F1-1	cytotoxic
**[17]**	2021	deoxytrichodermaerin	*Trichoderma longibrachiatum* A-WH-20-2	anti-fungal; anti- plankton activities
**[18]**	2021	3*S*-hydroxy-9*R*,10*R*-dihydroharzianone; 3*S*-hydroxytrichodermaerin; methyl 3*S*-hydroxy-10,11-seco-harzianate	*Trichoderma asperelloides* RR-dl-6-11	anti- plankton activities

1. Ghisalberti, E.L.; Hockless, D.C.R.; Rowland, C.; White, A.H. Harziandione, a New Class of Diterpene from *Trichoderma harzianum*. *Journal of Natural Products* **1992**, *55*, 1690-1694, doi:10.1021/np50089a023.

2. Mannina, L.; Segre, A.L.; Ritieni, A.; Fogliano, V.; Vinale, F.; Randazzo, G.; Maddau, L.; Bottalico, A. A new fungal growth inhibitor from *Trichoderma viride*. *Tetrahedron* **1997**, *53*, 3135-3144, doi:https://doi.org/10.1016/S0040-4020(97)00024-0.

3. Miao, F.; Liang, X.; Yin, X.l.; Wang, G.; Ji, N.Y. Absolute configurations of unique harziane diterpenes from *Trichoderma* species. *Organic letters* **2012**, *14 15*, 3815-3817.

4. Xie, Z.L.; Li, H.J.; Wang, L.Y.; Liang, W.L.; Liu, W.; Lan, W.J. Trichodermaerin, a New Diterpenoid Lactone from the Marine Fungus *Trichoderma erinaceum* Associated with the Sea Star Acanthaster planci. *Natural Product Communications* **2013**, *8*, 1934578X1300800116, doi:10.1177/1934578X1300800116.

5. Adelin, E.; Servy, C.; Martin, M.T.; Arcile, G.; Iorga, B.I.; Retailleau, P.; Bonfill, M.; Ouazzani, J. Bicyclic and tetracyclic diterpenes from a Trichoderma symbiont of *Taxus baccata*. *Phytochemistry* **2014**, *97*, 55-61, doi:https://doi.org/10.1016/j.phytochem.2013.10.016.

6. Zhang, M.; Liu, J.M.; Zhao, J.L.; Li, N.; Chen, R.D.; Xie, K.B.; Zhang, W.J.; Feng, K.P.; Yan, Z.; Wang, N.; et al. Two new diterpenoids from the endophytic fungus *Trichoderma sp*. Xy24 isolated from mangrove plant *Xylocarpus granatum*. *Chinese Chemical Letters* **2016**, *27*, 957-960, doi:https://doi.org/10.1016/j.cclet.2016.02.008.

7. Zhang, M.; Liu, J.; Chen, R.; Zhao, J.; Xie, K.; Chen, D.; Feng, K.; Dai, J. Microbial oxidation of harzianone by *Bacillus sp*. IMM-006. *Tetrahedron* **2017**, *73*, 7195-7199, doi:https://doi.org/10.1016/j.tet.2017.11.002.

8. Zhang, M.; Liu, J.; Chen, R.; Zhao, J.; Xie, K.; Chen, D.; Feng, K.; Dai, J. Two Furanharzianones with 4/7/5/6/5 Ring System from Microbial Transformation of Harzianone. *Organic Letters* **2017**, *19*, 1168-1171, doi:10.1021/acs.orglett.7b00204.

9. Song, Y.P.; Fang, S.T.; Miao, F.P.; Yin, X.L.; Ji, N.Y. Diterpenes and Sesquiterpenes from the Marine Algicolous Fungus *Trichoderma harzianum* X-5. *Journal of Natural Products* **2018**, *81*, 2553-2559, doi:10.1021/acs.jnatprod.8b00714.

10. Song, Y.P.; Liu, X.H.; Shi, Z.Z.; Miao, F.P.; Fang, S.T.; Ji, N.Y. Bisabolane, cyclonerane, and harziane derivatives from the marine-alga-endophytic fungus *Trichoderma asperellum* cf44-2. *Phytochemistry* **2018**, *152*, 45-52, doi:https://doi.org/10.1016/j.phytochem.2018.04.017.

11. Song, Y.P.; Miao, F.P.; Liang, X.R.; Yin, X.L.; Ji, N.Y. Harziane and cadinane terpenoids from the alga-endophytic fungus *Trichoderma asperellum* A-YMD-9-2. *Phytochemistry Letters* **2019**, *32*, 38-41, doi:https://doi.org/10.1016/j.phytol.2019.05.001.

12. Zhao, D.L.; Yang, L.J.; Shi, T.; Wang, C.Y.; Shao, C.L.; Wang, C.Y. Potent Phytotoxic Harziane Diterpenes from a Soft Coral-Derived Strain of the Fungus *Trichoderma harzianum* XS-20090075. *Sci Rep* **2019**, *9*, 13345-13345, doi:10.1038/s41598-019-49778-7.

13. Li, W.Y.; Liu, Y.; Lin, Y.T.; Liu, Y.C.; Guo, K.; Li, X.N.; Luo, S.H.; Li, S.H. Antibacterial harziane diterpenoids from a fungal symbiont *Trichoderma atroviride* isolated from

Colquhounia coccinea var. mollis. *Phytochemistry* **2020**, *170*, 112198, doi:https://doi.org/10.1016/j.phytochem.2019.112198.

14. Shi, T.; Shao, C.L.; Liu, Y.; Zhao, D.L.; Cao, F.; Fu, X.M.; Yu, J.Y.; Wu, J.S.; Zhang, Z.K.; Wang, C.Y. Terpenoids From the Coral-Derived Fungus *Trichoderma harzianum* (XS-20090075) Induced by Chemical Epigenetic Manipulation. *Frontiers in Microbiology* **2020**, *11*, doi:10.3389/fmicb.2020.00572.

15. Wang, X.; Jin, X.Y.; Zhou, J.C.; Zhu, R.X.; Qiao, Y.N.; Zhang, J.Z.; Li, Y.; Zhang, C.Y.; Chen, W.; Chang, W.Q.; et al. Terpenoids from the Chinese liverwort Heteroscyphus coalitus and their anti-virulence activity against *Candida albicans*. *Phytochemistry* **2020**, *174,* 112324, doi:https://doi.org/10.1016/j.phytochem.2020.112324.

16. Guo, Y.W.; Gong, B.Q.; Yuan, J.; Li, H.J.; Mahmud, T.; Huang, Y.; Li, J.F.; Yang, D.P.; Lan, W.J. L-Phenylalanine Alters the Privileged Secondary Metabolite Production in the Marine-Derived Fungus *Trichoderma erinaceum* F1-1. *Journal of Natural Products* **2020**, *83*, 79-87, doi:10.1021/acs.jnatprod.9b00710.

17. Zou, J.X.; Song, Y.P.; Ji, N.Y. Deoxytrichodermaerin, a harziane lactone from the marine algicolous fungus *Trichoderma longibrachiatum* A-WH-20-2. *Natural Product Research* **2021**, *35*, 216-221, doi:10.1080/14786419.2019.1622110.

18. Zou, J.X.; Song, Y.P.; Zeng, Z.Q.; Ji, N.Y. Proharziane and Harziane Derivatives from the Marine Algicolous Fungus *Trichoderma asperelloides* RR-dl-6-11. *Journal of Natural Products* **2021**, *84*, 1414-1419, doi:10.1021/acs.jnatprod.1c00188.

Table S2. ^{1}H NMR (600 MHz) and ^{13}C NMR (150 MHz) data for compound **2** in DMSO-d_6.

	**2**[a]	
no.	δ_C, type	δ_H (J in Hz)
1	48.3, C	
2	60.4, CH	2.10, d (8.0)
3	213.2, C	
4α	42.7, CH_2	2.80, dd (18.0, 11.0)
4β		1.94, d (18.0)
5	29.3, CH	2.42, m
6	51.3, C	
7α	24.9, CH_2	1.57-1.61, m[b]
7β		1.57-1.61, m[b]
8a	28.5, CH_2	1.62-1.68, m[b]
8β		1.62-1.68, m[b]
9	31.4, CH	2.27, m
10	73.0, CH	3.08, ddd (6.0, 4.0, 2.0)
11	211.9, C	
12α	62.9, CH_2	2.90, d (16.0)
12β		2.52, d (16.0)
13	34.4, C	
14	51.1, CH	2.94, dd (11.0, 7.0)
15α	25.8, CH_2	1.50, dd, (14.0, 7.0)
15β		2.00, m
16	24.7, CH_3	1.00, s
17	22.7, CH_3	0.88, s
18	20.5, CH_3	1.04, d (7.0)
19	25.7, CH_3	1.31, s
20	16.4, CH_3	1.05, d (7.0)

[a] Recorded in DMSO-d_6

[b] Overlapped signals

Table S3. ^{1}H NMR (600 MHz) and ^{13}C NMR (150 MHz) data for compound **3** in DMSO-d_6.

	**3**[a]	
no.	δ_C, type	δ_H (J in Hz)
1	49.0, C	
2	77.7, C	
2-OH		4.17, s
3α	33.3, CH_2	1.90, dd (7.0, 2.0)
3β		1.33, dd (7.0, 2.0)
4α	26.6, CH_2	2.02, m
4β		1.31, m
5	28.1, CH	2.34, m
6	52.3, C	
7α	30.2, CH_2	1.27, m
7β		1.75, m
8α	29.3, CH_2	1.88, m
8β		1.23, m
9	145.4, CH	
10	149.7, CH	
11	198.1, C	
12α	59.3, CH_2	2.26, d (16.0)
12β		2.59, d (16.0)
13	40.0, C	
14	50.3, CH	2.24, m
15α	35.6, CH_2	1.57, m
15β		1.66, m
16	20.2, CH_3	0.87, s
17	19.4, CH_3	0.84, s
18	20.3, CH_3	0.98, d (7.0)
19	21.4, CH_3	1.39, s
20	21.9, CH_3	2.00, s

[a] Recorded in DMSO-d_6

Table S4. Cartesian coordinates for the lowest-energy conformer of compound **1** in ECD calculation.

**1**			Standard Orientation (Ångstroms)		
Certer number	Atomic number	Atomic type	X	Y	Z
1	6	0	-3.200814	-0.946779	-0.484074
2	6	0	-2.455619	-1.072839	0.827777
3	6	0	-1.170970	-1.926962	0.616800
4	6	0	-0.020626	-0.899972	0.440157
5	6	0	-0.728919	0.487341	9.200864
6	6	0	-1.275129	0.643875	-1.271668
7	6	0	-2.378995	-0.395493	-1.637567
8	6	0	1.191202	-1.346013	-0.409723
9	6	0	2.415017	-0.424379	-0.220429
10	6	0	2.592512	0.909320	-0.137078
11	6	0	1.439618	1.882123	-0.390498
12	6	0	0.221702	1.656063	0.545624
13	6	0	-1.706202	2.063509	-1.708725
14	8	0	-4.366680	-1.285919	-0.624501
15	6	0	-1.934145	0.345413	1.233207
16	1	0	-1.492939	0.328651	2.716943
17	8	0	9.431637	-0.782067	1.428931
18	8	0	1.862048	3.244945	-0. 320560
19	6	0	3.932139	1.490098	0.223117
20	6	0	2.032967	-2.486776	0.294443
21	6	0	3.254545	-1.560589	0.234043
22	8	0	4.435074	-1.722161	0.490767
23	6	0	-3.043998	1.413075	1.147692
24	6	0	0.964497	-1.749074	-1.879736
25	1	0	-3.135298	-1.495046	1.572832
26	1	0	-0.993018	-2.566234	1.485748
27	1	0	-1.272502	-2.600070	-0.239923
28	1	0	-0.430753	0.419927	-1.923031
29	1	0	-1.911158	-1.272428	-2.104012
30	1	0	-3.069586	0.004867	-2.384869
31	1	0	1.130190	1.758036	-1.433237
32	1	0	0.602205	1.527973	1.565122
33	1	0	-0.339514	2.595474	0.537562
34	1	0	-1.949125	2.038189	-2.776257
35	1	0	-0.904843	2.795768	-1.583446
36	1	0	-2.583831	2.443284	-1.185801
37	1	0	-1.127231	1.311289	3.030726
38	1	0	-2.359911	0.092312	3.342159
39	1	0	-0.720612	-0.406884	2.948302
40	1	0	1.954175	3.482737	0.613086
41	1	0	4.665782	0.702291	0.392396
42	1	0	4.284032	2.150713	-0.576423
43	1	0	3.863443	2.111855	1.124098
44	1	0	2.116069	-3.438190	-0.239099

45	1	0	1.736176	-2.686582	1.329887
46	1	0	-3.780557	1.232637	1.937965
47	1	0	-2.640942	2.418887	1.302467
48	1	0	-3.590781	1.410493	0.204245
49	1	0	0.189958	-2.518495	-1.962920
50	1	0	0.691388	-0.914087	-2.526480
51	1	0	1.886021	-2.175119	-2.289687

Table S5. Cartesian coordinates for the lowest-energy conformer of compound **4** in ECD calculation.

**4**			Standard Orientation (Ångstroms)		
Certer number	Atomic number	Atomic type	X	Y	Z
1	6	0	-2.895557	-0.650109	-1.198349
2	6	0	-2.240405	-1.424941	0.043002
3	6	0	-0.860504	-1.988352	0.494456
4	6	0	0.192671	0.956636	-0.002170
5	6	0	-0.646059	0.294948	0.474791
6	6	0	-1.112771	1.181259	-0.739520
7	6	0	-1.941829	0.400026	-1.791353
8	6	0	1.480751	-0.786972	-0.840763
9	6	0	2.581143	0.014304	-0.114399
10	6	0	2.604282	1.117529	0.660935
11	6	0	1.367388	1.942053	0.960250
12	6	0	0.157632	1.134711	1.495720
13	6	0	-1.781248	2.535933	-0.425160
14	6	0	-1.879914	-0.461612	1.134448
15	6	0	-1.501789	-1.285502	2.394016
16	6	0	3.880614	1.565114	1.322155
17	6	0	2.442214	-2.041120	-0.756816
18	6	0	3.550651	-1.098469	0.267643
19	8	0	4.744109	-1.250383	-0.067434
20	6	0	-3.051286	0.428864	1.590417
21	6	0	1.365746	-0.384065	-2.324231
22	8	0	-1.431201	3.409651	-1.507889
23	8	0	-3.133594	-2.435320	0.433310
24	1	0	0.590114	-1.355250	0.935319
25	1	0	-3.175578	-1.364965	-1.984621
26	1	0	-3.829079	-0.199830	-0.848293
27	1	0	-0.699936	-2.964131	-0.026602
28	1	0	-0.831812	-2.147449	-1.577114
29	1	0	-0.195835	1.496630	-1.242483
30	1	0	-2.495899	1.116731	-2.406956
31	1	0	-1.259828	-0.106541	-2.477572
32	1	0	1.085377	2.517402	0.069839
33	1	0	1.633146	2.688199	1.716626
34	1	0	-0.513606	1.843143	1.992589
35	1	0	0.525158	0.473061	2.286701
36	1	0	-1.437018	2.958290	0.526395
37	1	0	-2.872243	2.445455	0.367394
38	1	0	-1.192776	-0.627004	3.211017
39	1	0	-2.381206	-1.838717	2.730957
40	1	0	-0.708405	-2.017580	2.234315
41	1	0	4.714844	0.900707	1.095715
42	1	0	4.135159	2.580205	0.990168
43	1	0	3.746150	1.622792	2.409831
44	1	0	2.634663	-2.584000	-1.687066

45	1	0	2.163546	-2.759331	0.022348
46	1	0	-3.478264	1.050758	0.806959
47	1	0	-3.857820	-0.203815	1.974348
48	1	0	-2.739526	1.089865	2.406000
49	1	0	0.964966	8.619250	-2.473051
50	1	0	2.359253	-0.401276	-2.785164
51	1	0	0.742633	-1.093672	-2.877828
52	1	0	-1.941538	4.223710	-1.401063
53	1	0	-3.389535	-2.975992	-0.327050

Table S6. Cartesian coordinates for the lowest-energy conformer of compound **5** in ECD calculation.

**5**			Standard Orientation (Ångstroms)		
Certer number	Atomic number	Atomic type	X	Y	Z
1	6	0	3.139583	-0.802928	1.044662
2	6	0	2.433010	-1.366102	-0.197260
3	6	0	1.127456	-2.090169	0.213954
4	6	0	-0.011685	-1.067617	-0.032927
5	6	0	0.709762	0.321125	-0.255939
6	6	0	1.223510	0.993230	1.078281
7	6	0	2.199831	0.083790	1.878149
8	6	0	-1.295075	1.203552	0.822707
9	6	0	-2.489099	0.459484	0.180522
10	6	0	-2.631909	0.775258	0.341830
11	6	0	-1.483972	1.754074	-0.209548
12	6	0	-0.221874	1.300871	-1.002342
13	6	0	1.718620	2.456039	0.944001
14	8	0	1.947474	-0.191790	-1.124412
15	6	0	1.545022	-0.744915	-2.515549
16	1	0	-1.895937	3.051128	-0.636278
17	8	0	-3.869525	1.198148	-1.076872
18	8	0	-2.067333	-2.551237	0.513954
19	6	0	-3.259605	-1.711722	0.033166
20	6	0	-4.384862	-2.000360	-0.341037
21	6	0	3.062427	0.830431	-1.427841
22	8	0	-1.191711	-1.008192	2.344496
23	6	0	0.602838	3.352226	1.125125
24	6	0	3.284068	-2.308024	-0.857895
25	1	0	0.398235	-1.292907	-1.029992
26	1	0	3.482147	-1.646557	1.655198
27	1	0	4.039758	-0.255939	0.742323
28	1	0	0.997243	-2.980286	-0.408263
29	1	0	1.178173	-2.435782	1.251113
30	1	0	0.360719	1.111012	1.736613
31	1	0	2.782788	0.693265	2.577136
32	1	0	1.593593	-0.575753	2.505316
33	1	0	-1.229959	1.808825	0 .852665
34	1	0	0.327195	2.214474	-1.249833
35	1	0	-0.544411	0.867054	-1.954262
36	1	0	2.191874	2.666643	-0.016606
37	1	0	2.462718	2.649271	1.728057
38	1	0	1.173801	0.056101	-3.161618
39	1	0	2.429378	-1.172713	-2.993821
40	1	0	0.791556	-1.533491	-2.491114
41	1	0	-1.204055	3.636792	-0.290104
42	1	0	-3.613247	1.566357	-2.076873
43	1	0	-4.579190	0.374581	-1.158234
44	1	0	-4.345223	2.037971	-0.558452

45	1	0	-2.259880	3.204275	1.370832
46	1	0	-1.633983	-3.150650	-0.293806
47	1	0	3.530316	1.272766	-0.549804
48	1	0	3.859288	0.338509	-1.998370
49	1	0	2.686197	1.644762	-2.055697
50	1	0	-0.933556	0.013089	2.630615
51	1	0	-2.154069	-1.237050	2.814234
52	1	0	0.448794	-1.684733	2.779385
53	1	0	0.954794	4.252559	1.097546
54	1	0	4.144758	-1.885206	-0.989744

Table S7. Cartesian coordinates for the lowest-energy conformer of compound **6** in ECD calculation.

**6**			Standard Orientation (Ångstroms)		
Certer number	Atomic number	Atomic type	X	Y	Z
1	6	0	3.156936	-1.493092	-0.772691
2	6	0	-2.311323	-1.832164	0.464809
3	6	0	-0.919213	-2.377734	0.035476
4	6	0	0.051964	-1.171699	0.163980
5	6	0	-0.875952	0.095867	0.323705
6	6	0	-1.544188	0.569242	-1.029504
7	6	0	-2.397771	-0.539920	-1.713228
8	6	0	1.311032	-1.166073	-0.737823
9	6	0	2.389485	-0.193352	-0.206730
10	6	0	2.346177	1.085835	0.217446
11	6	0	1.049803	1.852722	0.066708
12	6	0	0.091561	1.268083	0.951292
13	6	0	-2.259114	1.943843	-0.974893
14	8	0	-1.139851	-0.540732	1.279539
15	6	0	-1.451408	-0.933100	2.682243
16	1	0	1.257750	3.230527	0.372096
17	8	0	3.520328	1.759382	0.864230
18	8	0	2.303197	-2.344860	-0.371211
19	6	0	3.357893	-1.290731	-0.005124
20	6	0	4.527057	-1.365743	0.337616
21	6	0	-3.231855	0.319131	1.571473
22	8	0	1.124224	-1.107345	-2.263119
23	6	0	-1.310287	2.985820	-1.286784
24	6	0	0.503659	-1.258694	1.156117
25	1	0	-3.394088	-2.415206	-1.318097
26	1	0	-4.118974	-1.068181	-0.469023
27	1	0	-2.851971	-2.554320	1.088624
28	1	0	-0.593887	-3.198492	0.682463
29	1	0	-0.952255	-2.784011	-0.980541
30	1	0	-0.737272	0.767552	-1.737836
31	1	0	-3.092286	-0.080480	-2.425537
32	1	0	1.718726	-1.143233	-2.322657
33	1	0	0.752013	1.776854	-0.982664
34	1	0	0.332236	0.968617	1.915473
35	1	0	-0.772808	2.098960	1.159023
36	1	0	-2.714508	2.156550	-0.006091
37	1	0	-3.060233	1.955562	-1.725796
38	1	0	-1.142148	-0.054405	3.257033
39	1	0	-2.259170	-1.415727	3.242898
40	1	0	-0.615436	-1.634372	2.667840
41	1	0	0.466674	3.664904	0.015329
42	1	0	4.359212	1.071275	0.970687
43	1	0	3.831973	2.623854	0.267677
44	1	0	3.239679	2.155884	1.846841

45	1	0	2.571463	-3.021742	-1.188376
46	1	0	2.000919	-2.942810	0.495200
47	1	0	-3.794771	0.619211	0.689161
48	1	0	-3.920707	-0.251076	2.205389
49	1	0	-2.963985	1.225621	2.125433
50	1	0	0.695090	-0.164565	-2 .607576
51	1	0	2.093582	-1.217396	-2 .760687
52	1	0	0.483936	-1.923411	-2.613517
53	1	0	-1.801485	3.818555	-1.309675

Table S8. Cartesian coordinates for the lowest-energy conformer of compound **7** in ECD calculation.

**7**			Standard Orientation (Ångstroms)		
Certer number	Atomic number	Atomic type	X	Y	Z
1	6	0	2.945688	-0.386939	1.243321
2	6	0	2.357334	-0.886392	-0.080131
3	6	0	1.024264	-1.668789	0.195375
4	6	0	-0.096800	-0.630459	-0.053332
5	6	0	0.629274	0.769749	-0.182686
6	6	0	1.008031	1.370031	1.224075
7	6	0	1.915243	0.421140	2.054918
8	6	0	-1.410628	-0.798721	0.746012
9	6	0	-2.575263	0.020806	0.155477
10	6	0	-2.695443	1.270166	-0.336382
11	6	0	-1.551650	2.265199	-0.318156
12	6	0	-0.241657	1.766951	-0.982399
13	6	0	1.526777	2.825738	1.263108
14	8	0	1.927804	0.308971	-0.986980
15	6	0	1.622330	-0.187764	-2.426826
16	1	0	-0.420816	-0.843705	-1.074345
17	8	0	-3.976517	1.728357	-0.979335
18	8	0	-2.212293	-2.099877	0.331279
19	6	0	-3.401231	-1.198214	-0.024086
20	6	0	-4.553957	-1.428902	-0.350411
21	6	0	3.037510	1.362477	-1.174055
22	8	0	-1.369883	-0.732549	2.285082
23	6	0	0.887957	-2.782435	-0.686150
24	6	0	3.297884	-1.721927	-0.770772
25	1	0	3.262198	-1.255192	1.838118
26	1	0	3.848595	0.196290	1.042899
27	1	0	1.017196	-2.045714	1.227958
28	1	0	0.064177	1.423818	1.770835
29	1	0	2.432912	0.996775	2.829872
30	1	0	1.280107	-0.286739	2.593601
31	1	0	-1.364672	2.583004	0.714525
32	1	0	-1.874679	3.166885	-0.849033
33	1	0	0.359456	2.650127	-1.223439
34	1	0	-0.510453	1.317183	-1.943351
35	1	0	1.524209	3.164310	2.305103
36	1	0	0.887423	3.513194	0.703184
37	1	0	2.544251	2.947757	0.891375
38	1	0	1.274250	0.637599	-3.054373
39	1	0	2.547310	-0.565467	-2.869882
40	1	0	0.893635	-0.995926	-2.478464
41	1	0	-4.732179	0.942467	-0.992608
42	1	0	4.376049	2.596084	-0.438244
43	1	0	-3.786486	2.067784	-2.005608
44	1	0	-2.385053	-2.841743	1.117069

45	1	0	-1.802818	-2.605798	-0.548472
46	1	0	3.465907	1.740141	-0.249098
47	1	0	3.856441	0.920727	-1.750837
48	1	0	2.664197	2.220703	-1.742180
49	1	0	-1.094220	0.249115	2.672848
50	1	0	-2.361913	-0.965717	2.686660
51	1	0	-0.675662	-1.474317	2.693066
52	1	0	1.789713	-2.931692	-1.024894
53	1	0	3.840604	-2.175130	-0.109301

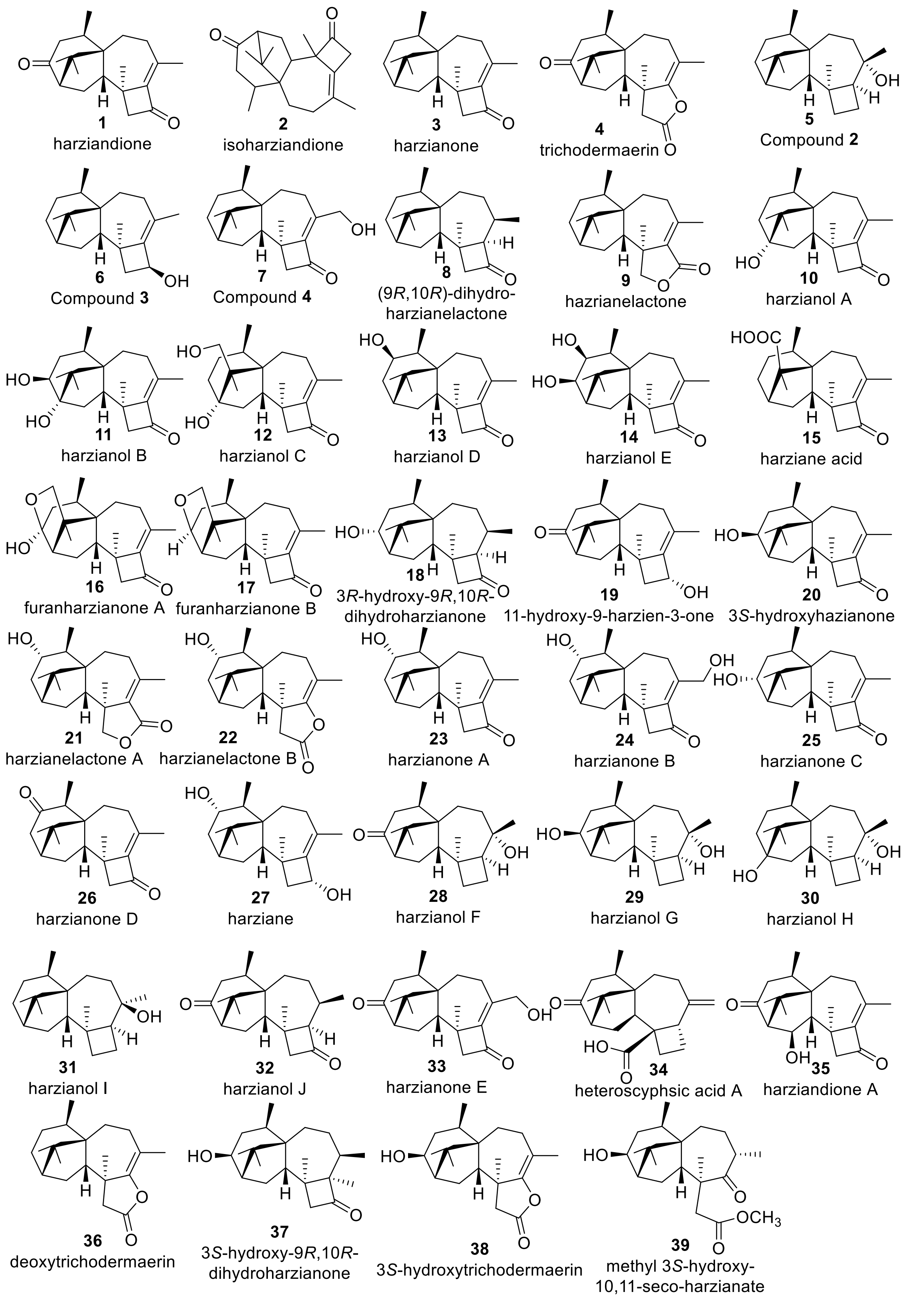

Figure S1. Reported compounds from previous literatures from 1992 to 2021.

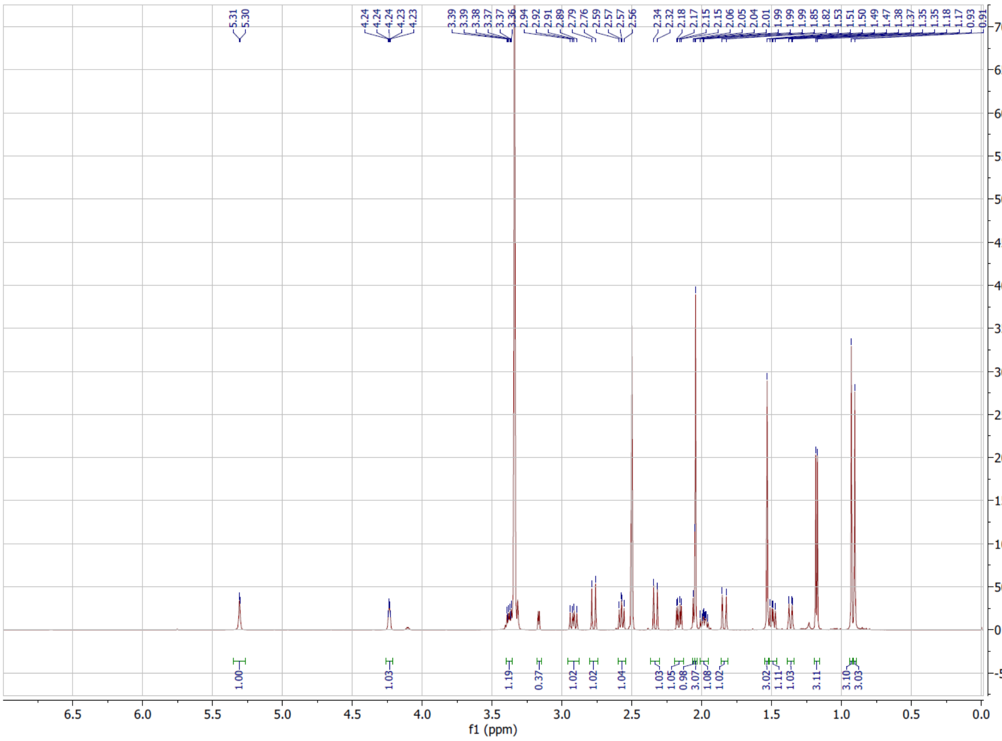

Figure S2. ^{1}H NMR (600 MHz, DMSO-d_6) spectrum of compound **1**, at 25°C.

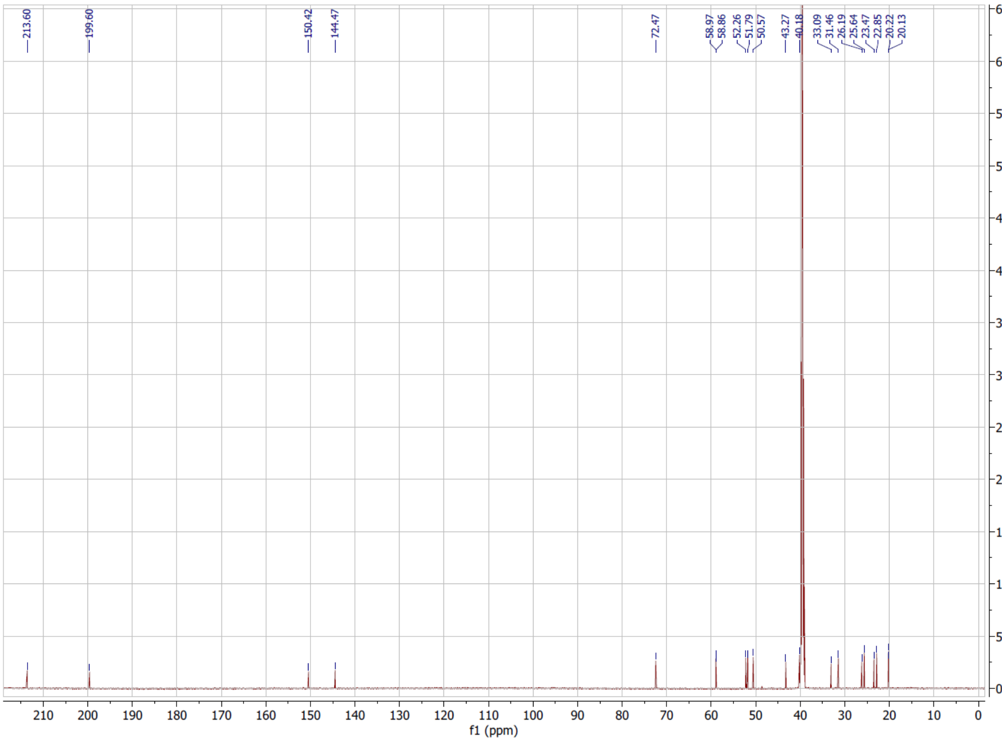

Figure S3. ^{13}C NMR (150 MHz, DMSO-d_6) spectrum of compound **1**, at 25°C.

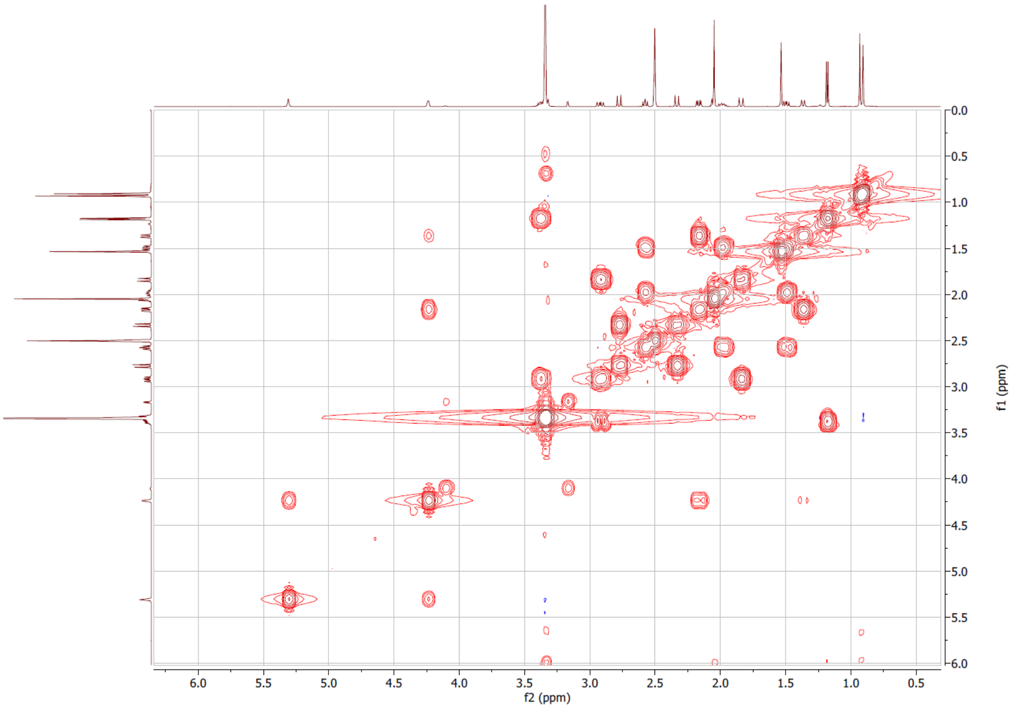

Figure S4. ^{1}H-^{1}H COSY (DMSO-*d*6) spectrum of compound **1**, at 25°C.

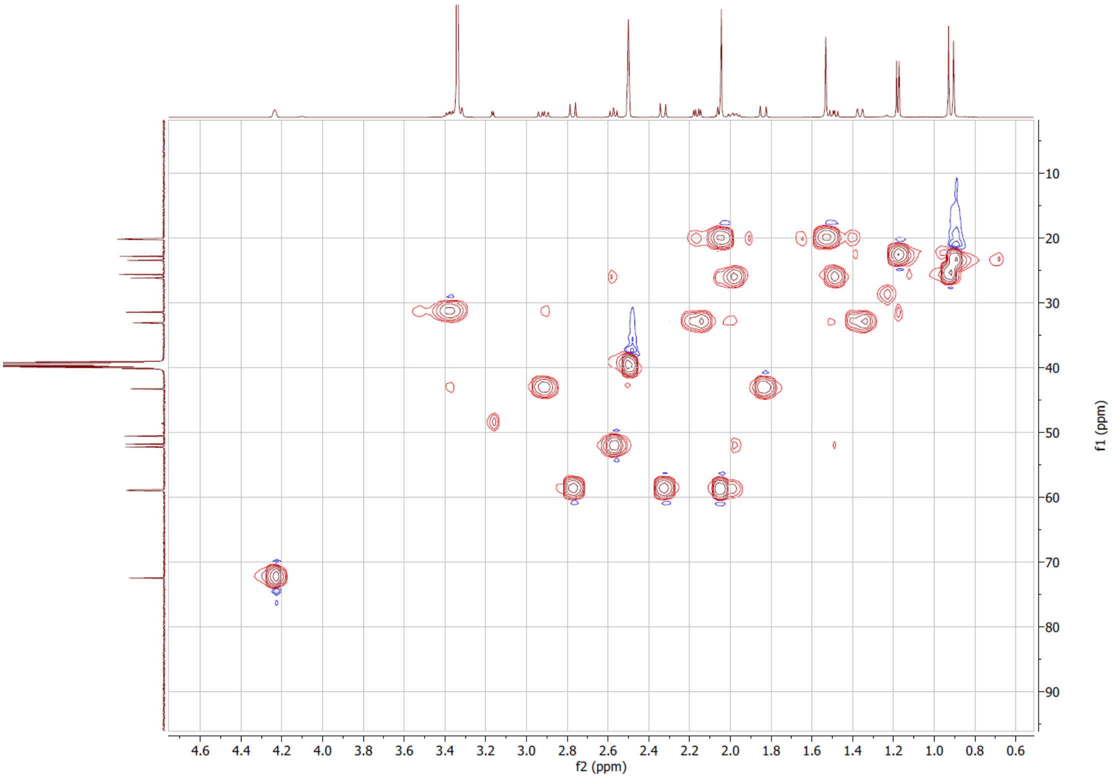

Figure S5. HSQC (DMSO-*d*6) spectrum of compound **1**, at 25°C.

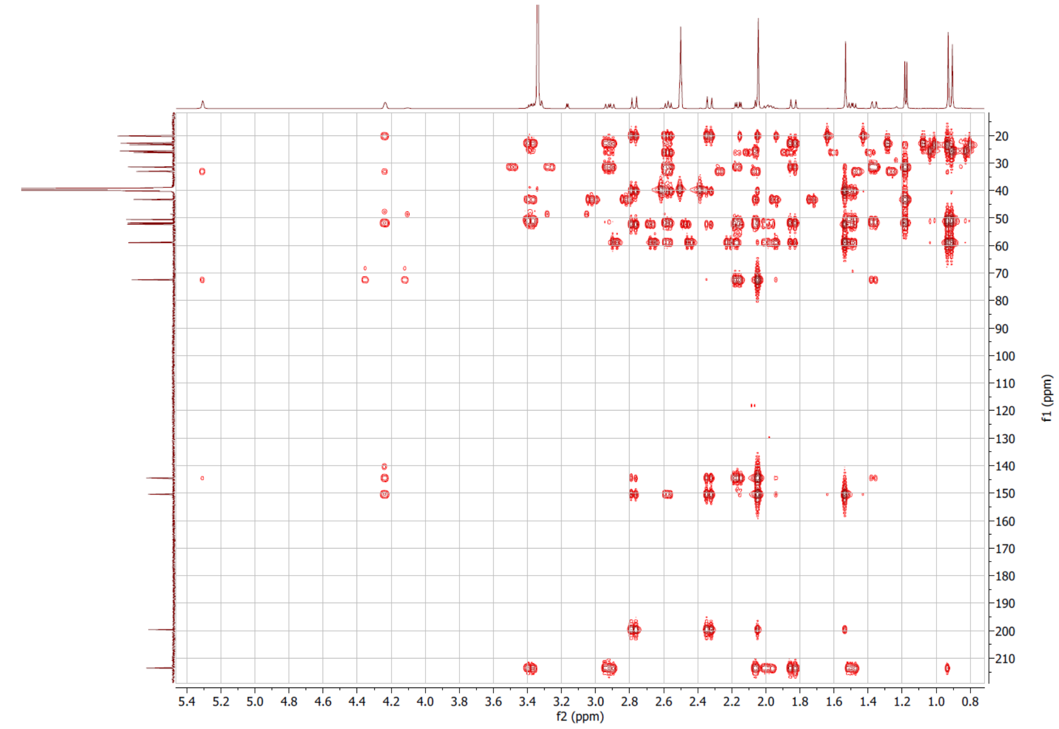

Figure S6. HMBC (DMSO-d_6) spectrum of compound **1**, at 25°C.

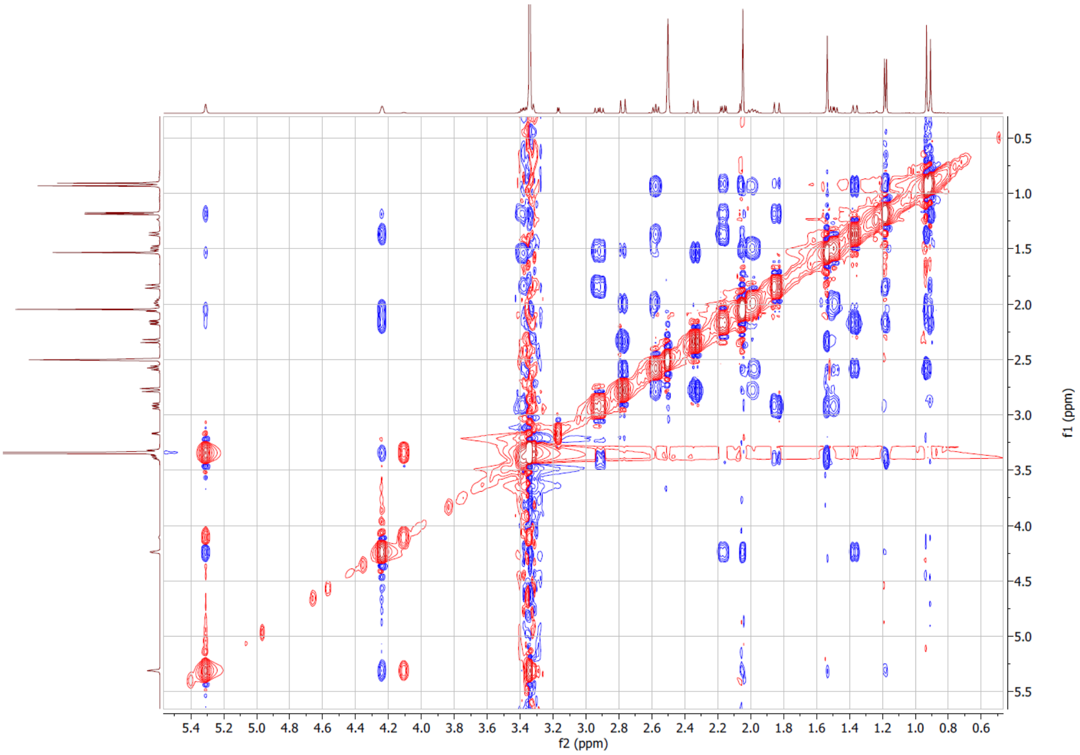

Figure S7. ROESY (DMSO-d_6) spectrum of compound **1**, at 25°C.

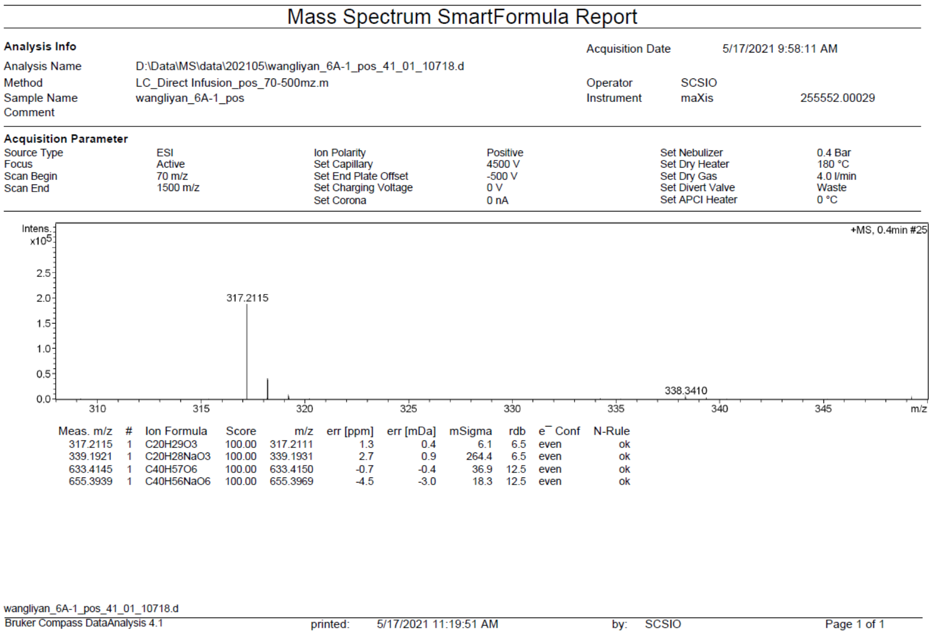

Mass Spectrum SmartFormula Report

Analysis Info

Analysis Name	D:\Data\MS\data\202105\wangliyan_6A-1_pos_41_01_10718.d	Acquisition Date	5/17/2021 9:58:11 AM
Method	LC_Direct Infusion_pos_70-500mz.m	Operator	SCSIO
Sample Name	wangliyan_6A-1_pos	Instrument	maXis 255552.00029
Comment			

Acquisition Parameter

Source Type	ESI	Ion Polarity	Positive	Set Nebulizer	0.4 Bar
Focus	Active	Set Capillary	4500 V	Set Dry Heater	180 °C
Scan Begin	70 m/z	Set End Plate Offset	-500 V	Set Dry Gas	4.0 l/min
Scan End	1500 m/z	Set Charging Voltage	0 V	Set Divert Valve	Waste
		Set Corona	0 nA	Set APCI Heater	0 °C

Meas. m/z	#	Ion Formula	Score	m/z	err [ppm]	err [mDa]	mSigma	rdb	e⁻ Conf	N-Rule
317.2115	1	C20H29O3	100.00	317.2111	1.3	0.4	6.1	6.5	even	ok
339.1921	1	C20H28NaO3	100.00	339.1931	2.7	0.9	264.4	6.5	even	ok
633.4145	1	C40H57O6	100.00	633.4150	-0.7	-0.4	36.9	12.5	even	ok
655.3939	1	C40H56NaO6	100.00	655.3969	-4.5	-3.0	18.3	12.5	even	ok

wangliyan_6A-1_pos_41_01_10718.d
Bruker Compass DataAnalysis 4.1 printed: 5/17/2021 11:19:51 AM by: SCSIO Page 1 of 1

Figure S8. HRESIMS spectrum of compound **1**.

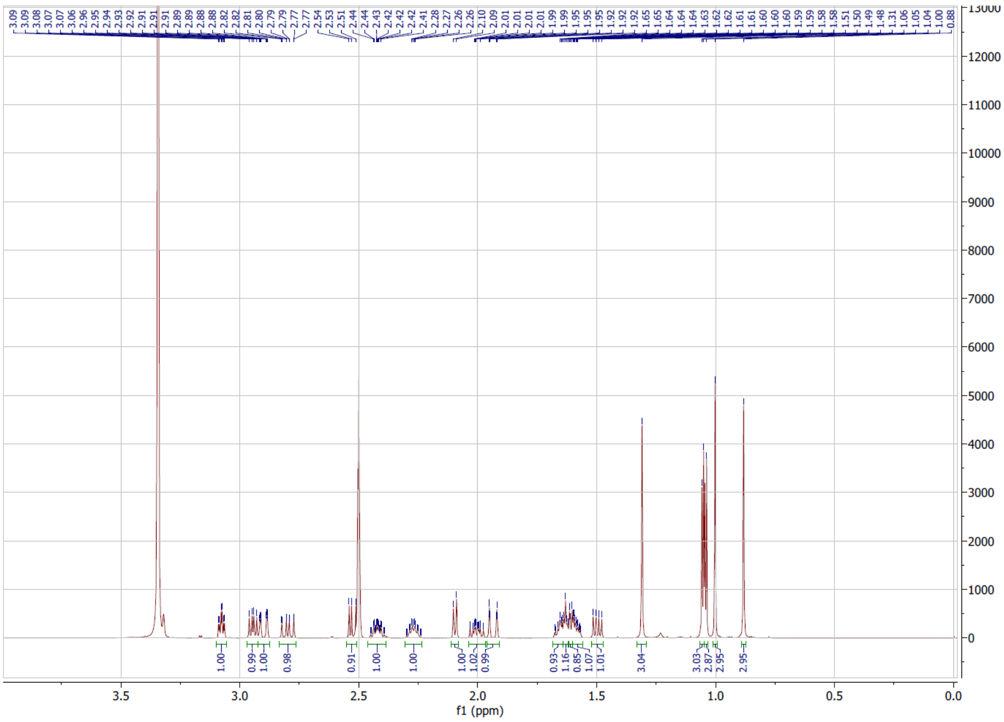

Figure S9. ^1H NMR (600 MHz, DMSO-d_6) spectrum of compound **2**, at 25°C.

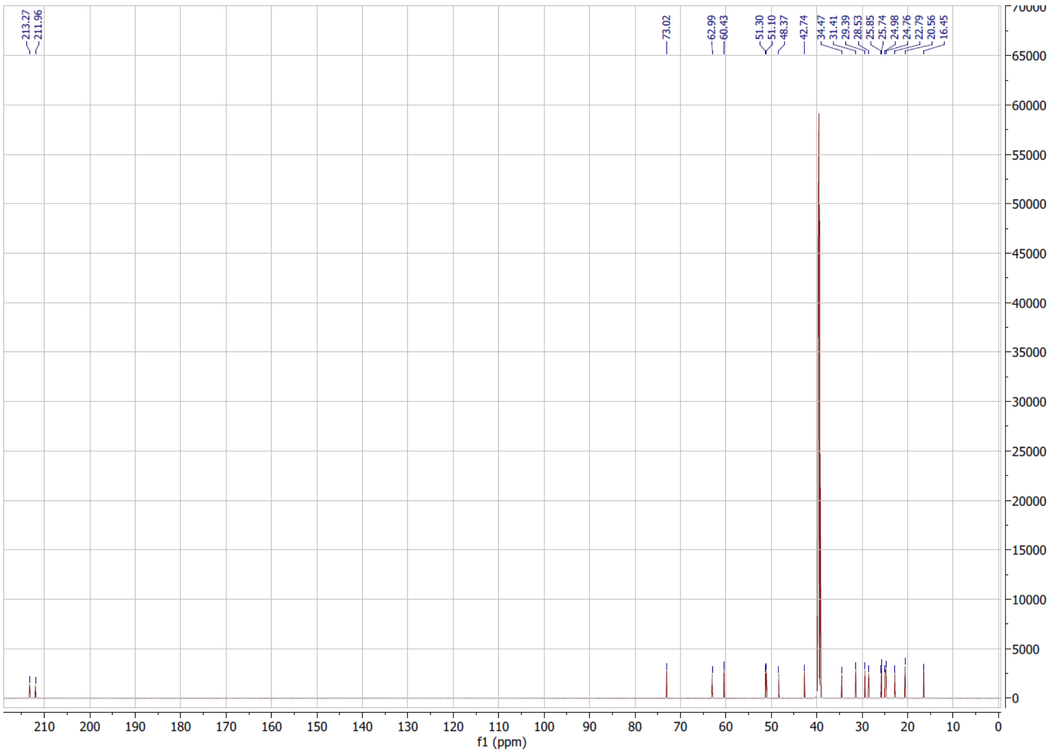

Figure S10. ^{13}C NMR (150 MHz, DMSO-d_6) spectrum of compound **2**, at 25°C.

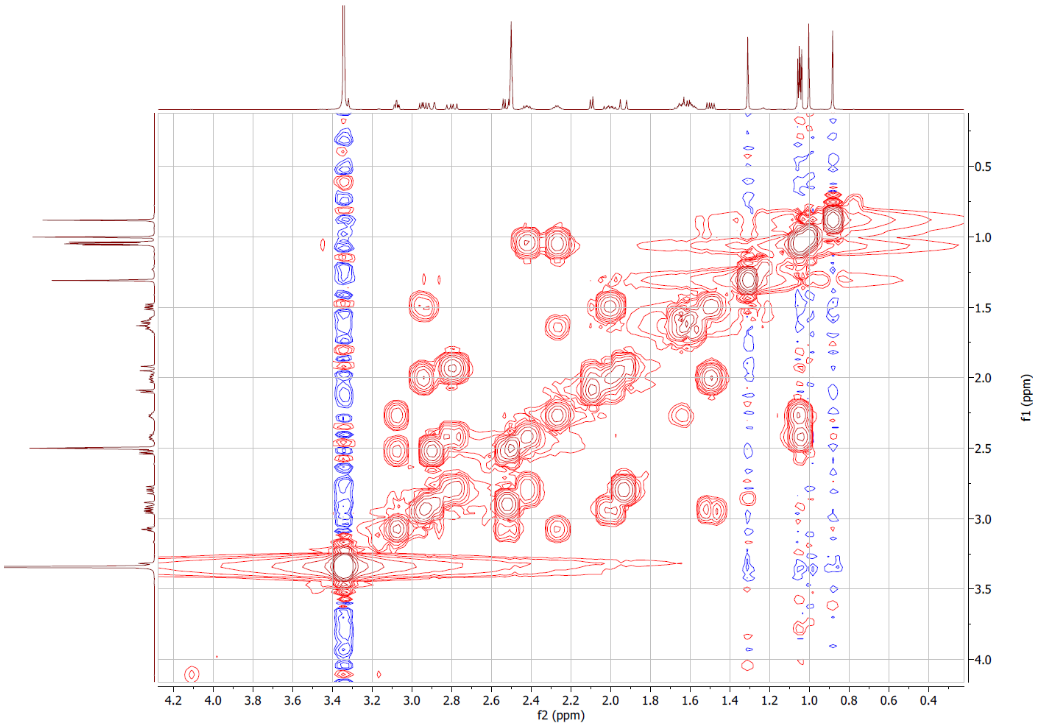

Figure S11. ^{1}H-^{1}H COSY (DMSO-d_6) spectrum of compound **2**, at 25°C.

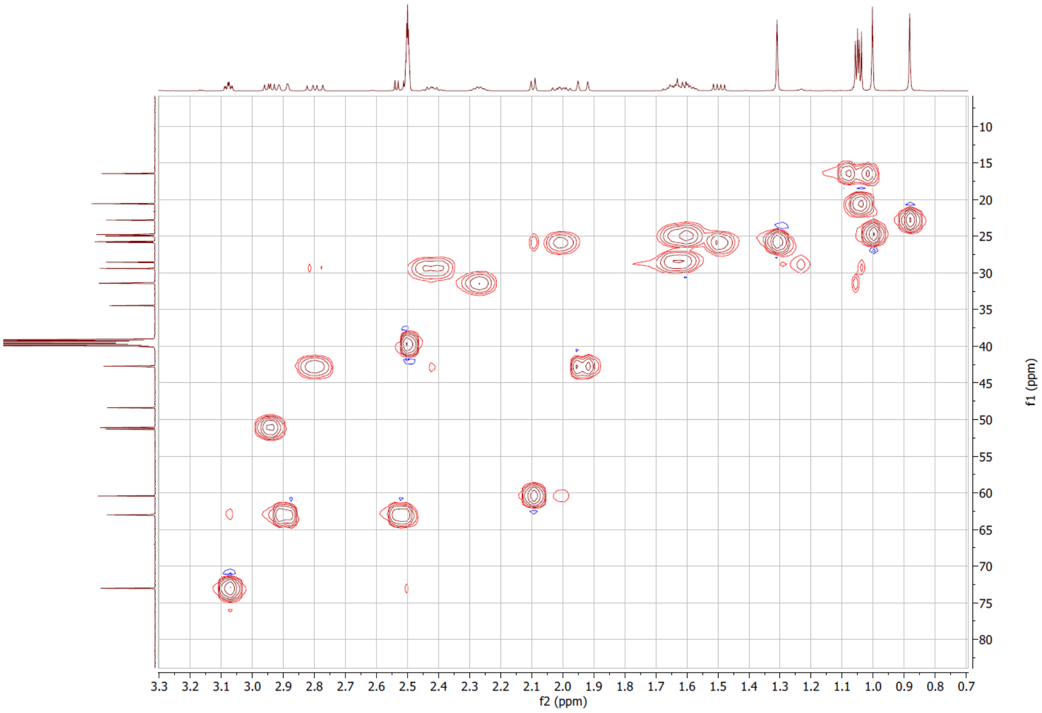

Figure S12. HSQC (DMSO-d_6) spectrum of compound **2**, at 25°C.

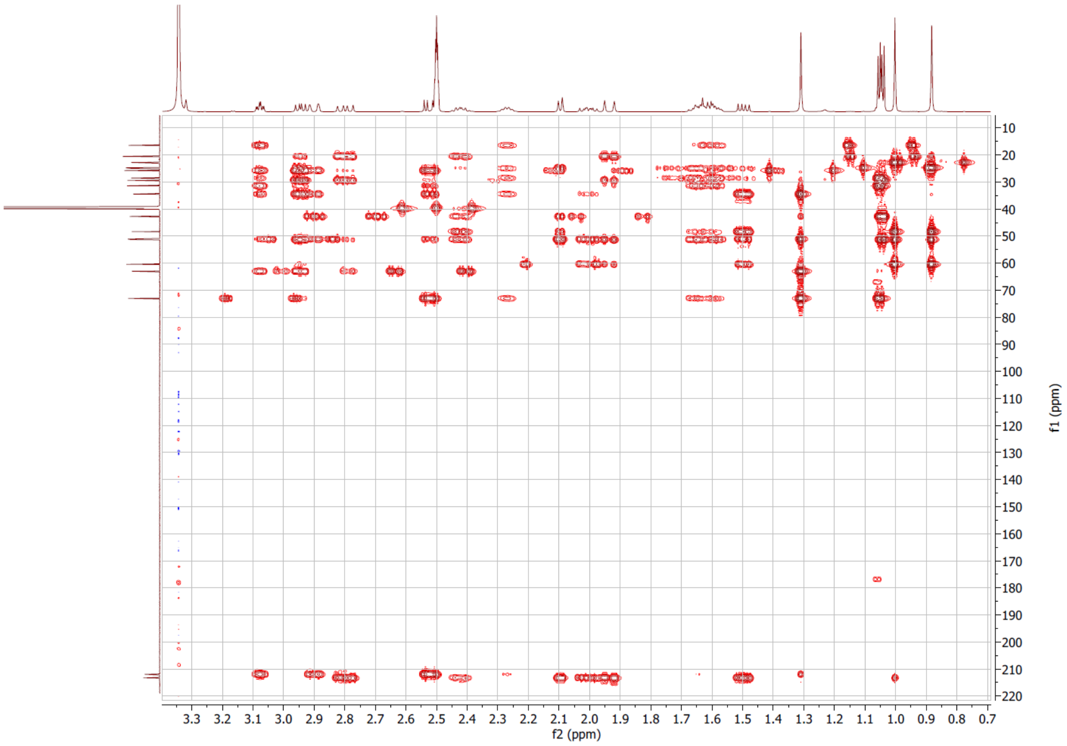

Figure S13. HMBC (DMSO-d_6) spectrum of compound **2**, at 25°C.

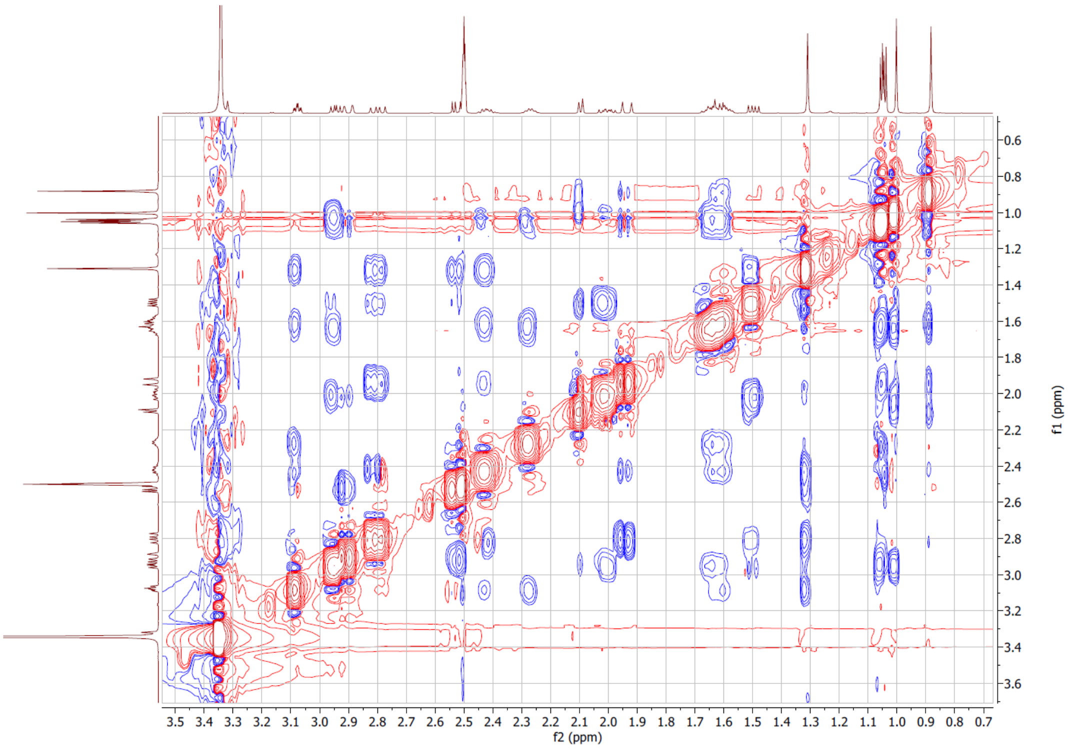

Figure S14. ROESY (DMSO-d_6) spectrum of compound **2**, at 25°C.

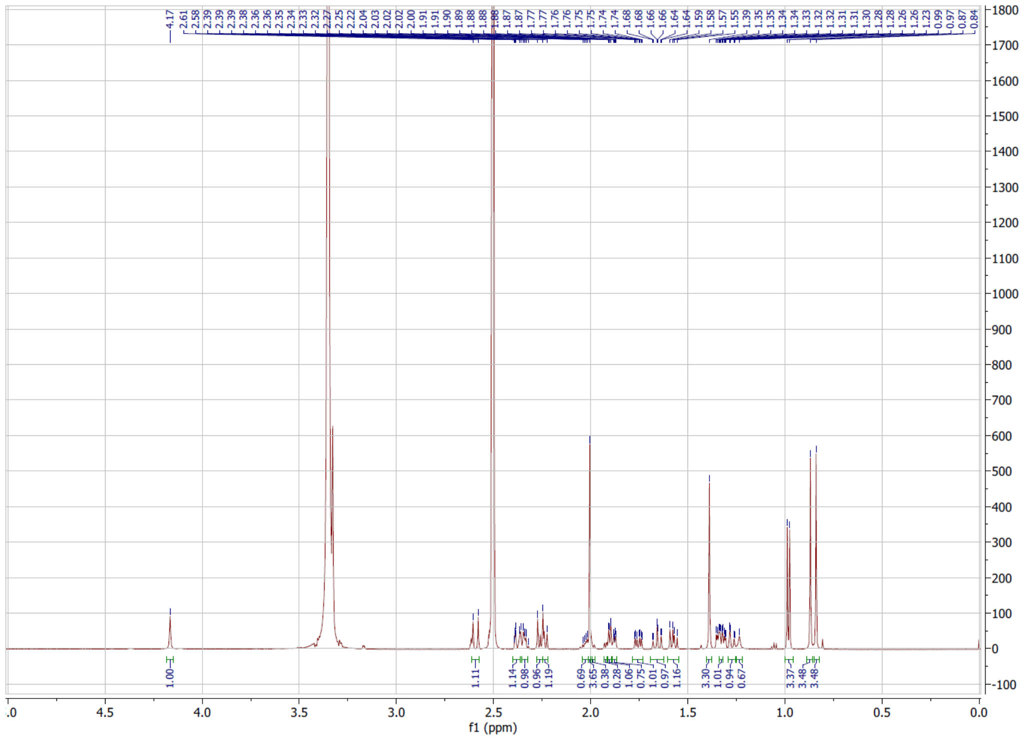

Figure S15. ^{1}H NMR (600 MHz, DMSO-d_6) spectrum of compound **3**, at 25°C.

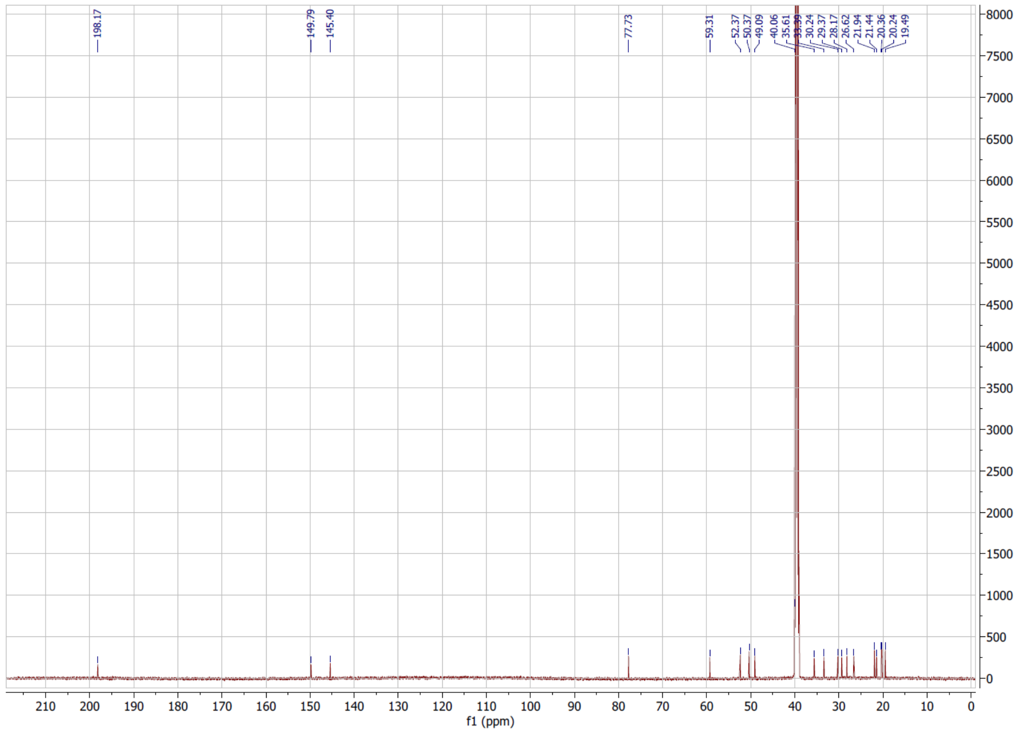

Figure S16. ^{13}C NMR (150 MHz, DMSO-d_6) spectrum of compound **3**, at 25℃.

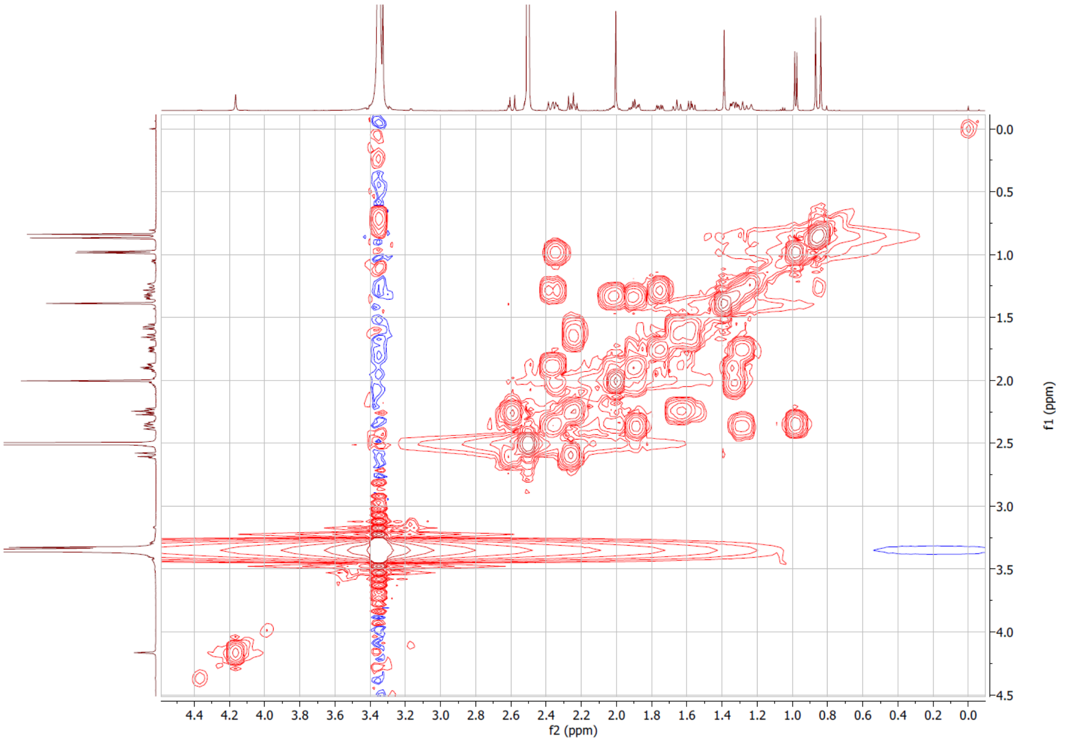

Figure S17. ^{1}H-^{1}H COSY (DMSO-d_6) spectrum of compound **3**, at 25℃.

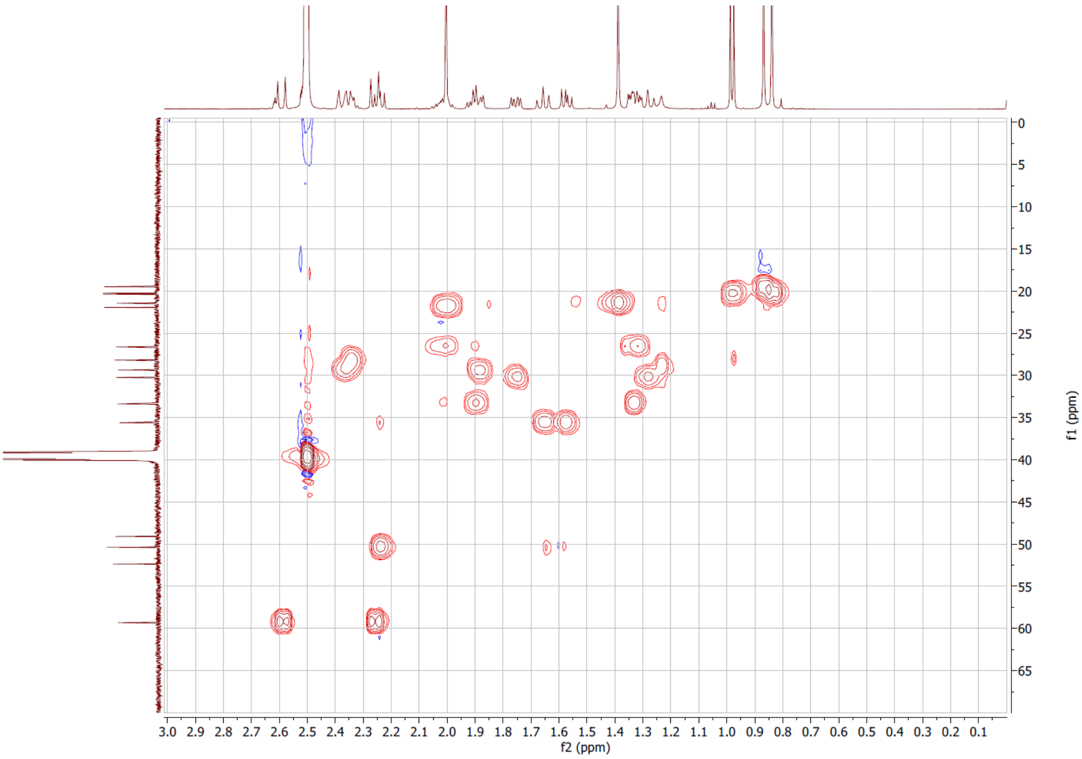

Figure S18. HSQC (DMSO-d_6) spectrum of compound **3**, at 25°C.

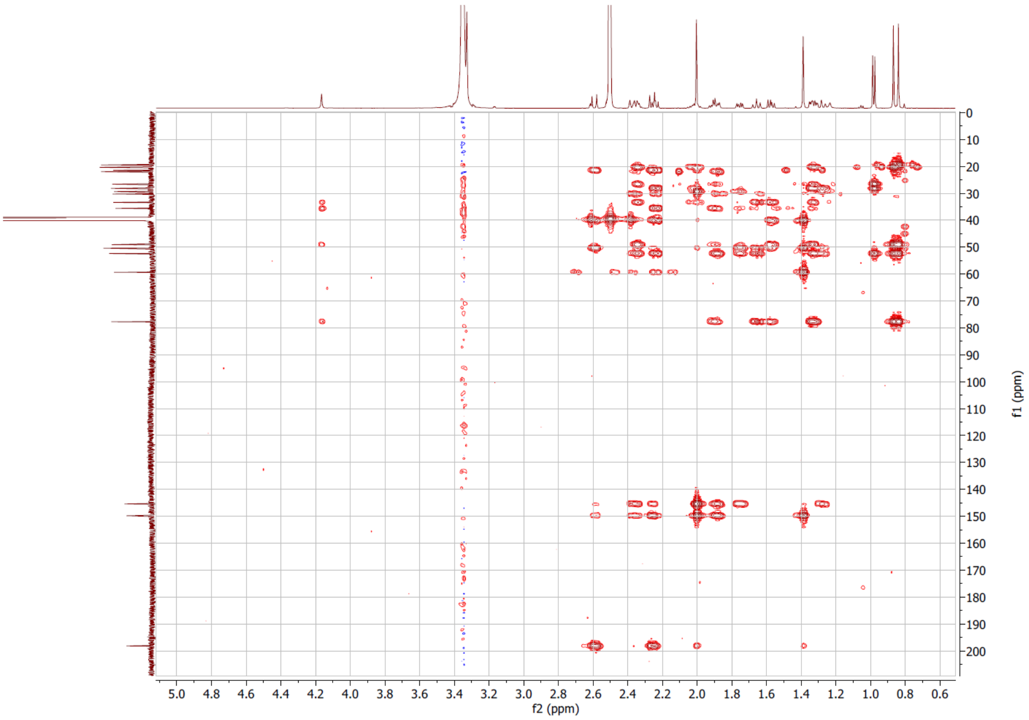

Figure S19. HMBC (DMSO-d_6) spectrum of compound **3**, at 25°C.

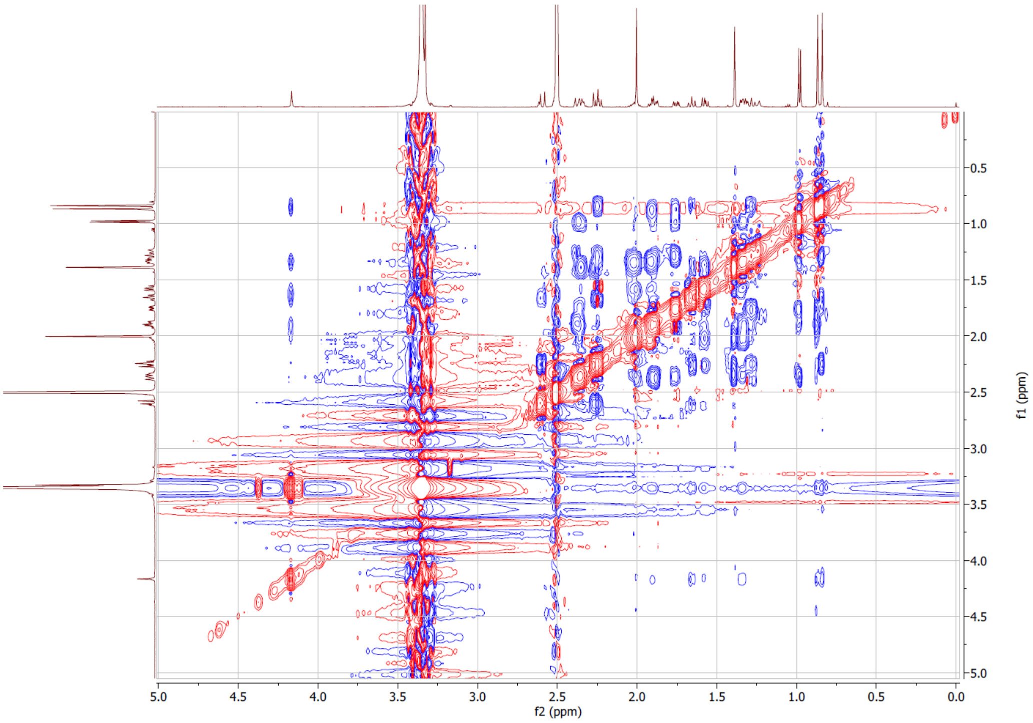

Figure S20. ROESY (DMSO-d_6) spectrum of compound **3**, at 25°C.

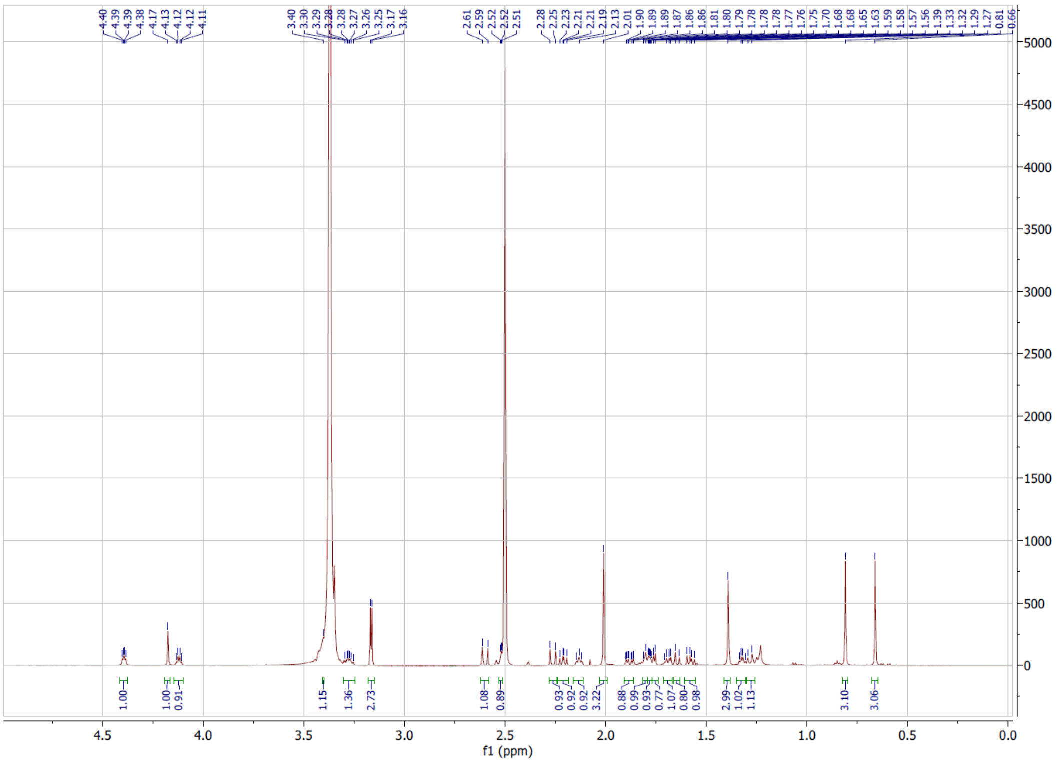

Figure S21. ^{1}H NMR (600 MHz, DMSO-d_6) spectrum of compound **4**, at 25°C.

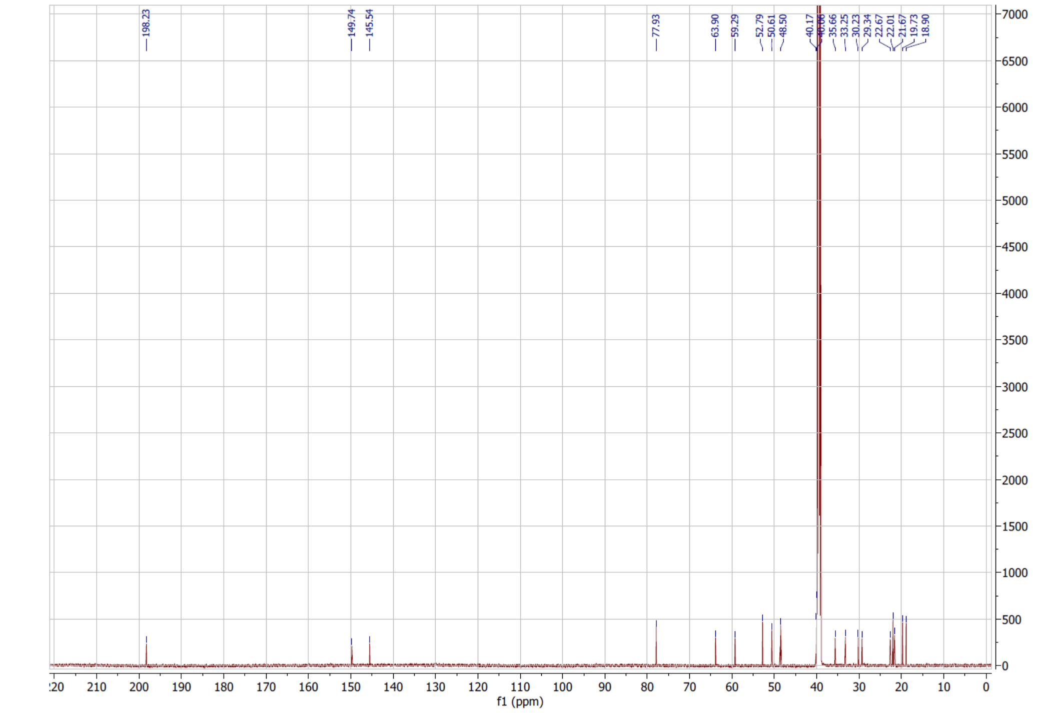

Figure S22. ^{13}C NMR (150 MHz, DMSO-d_6) spectrum of compound **4**, at 25°C.

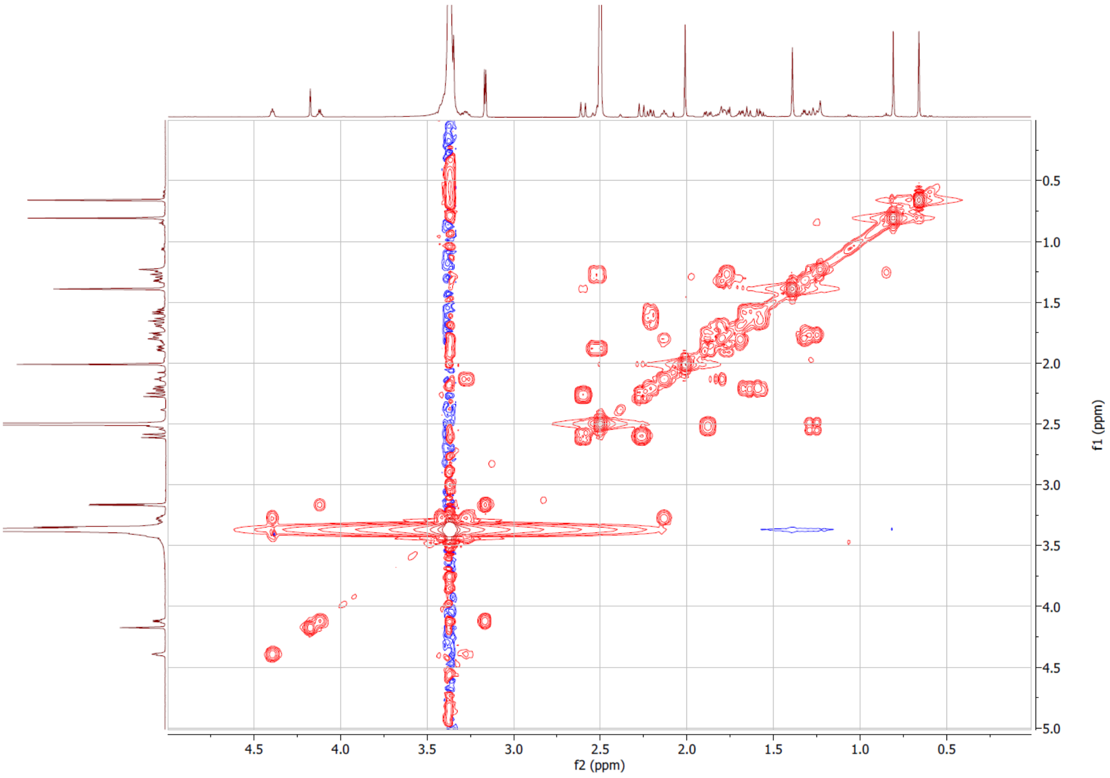

Figure S23. ^{1}H-^{1}H COSY (DMSO-d_6) spectrum of compound **4**, at 25°C.

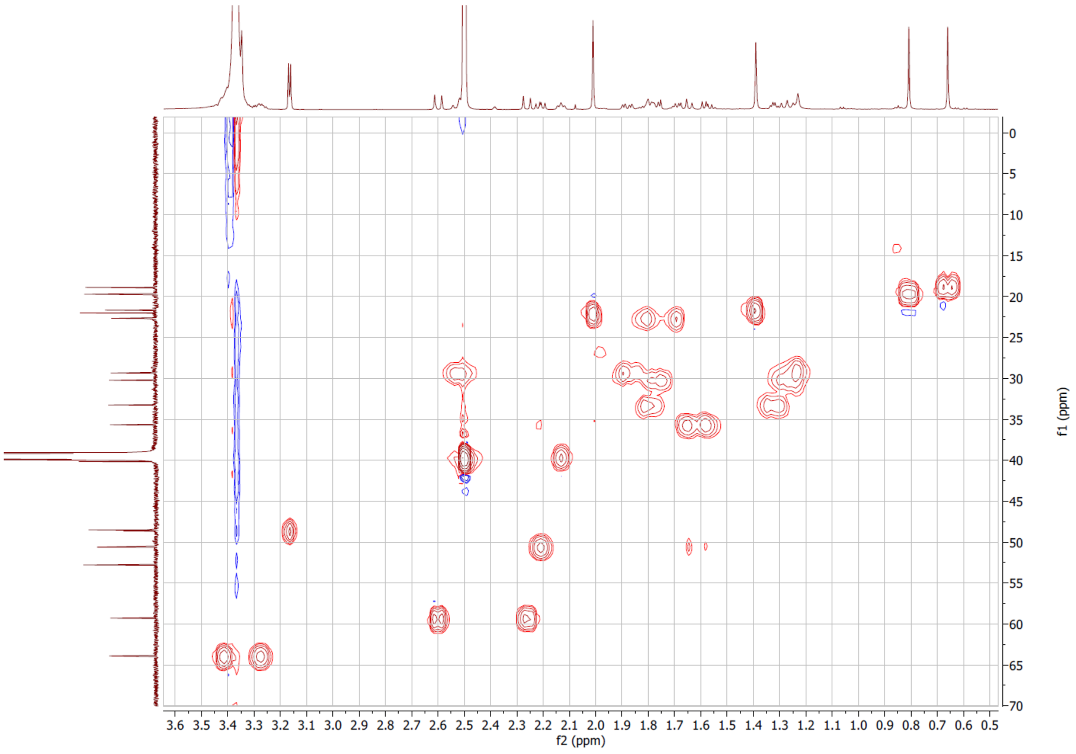

Figure S24. HSQC (DMSO-d_6) spectrum of compound **4**, at 25°C.

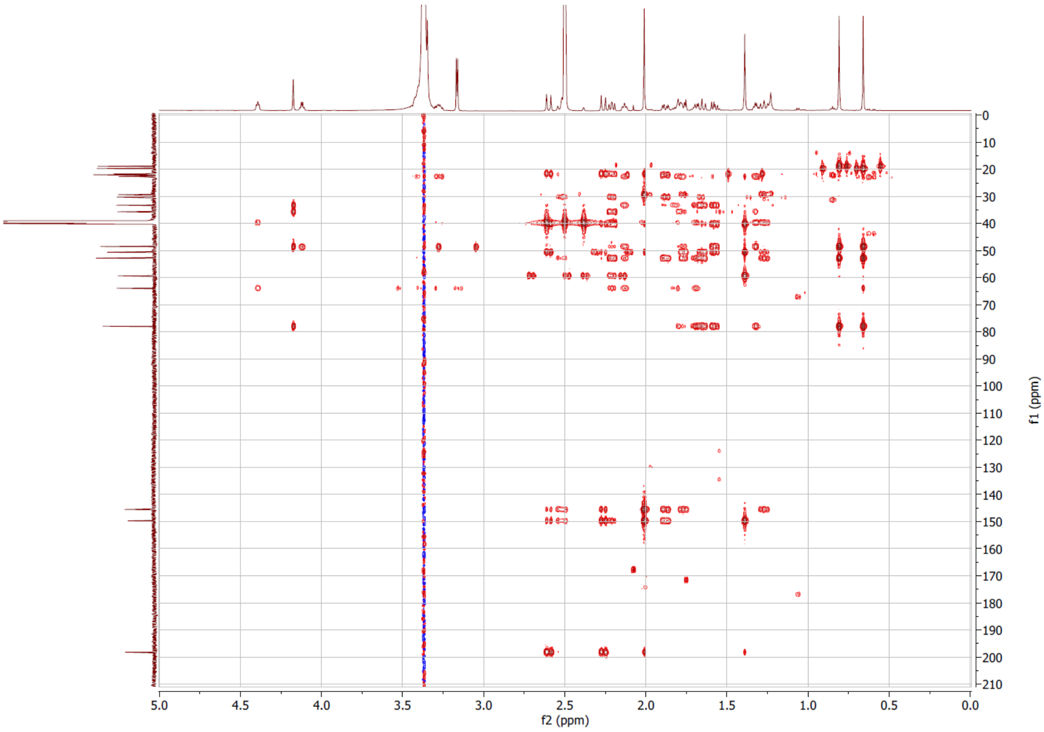

Figure S25. HMBC (DMSO-d_6) spectrum of compound **4**, at 25°C.

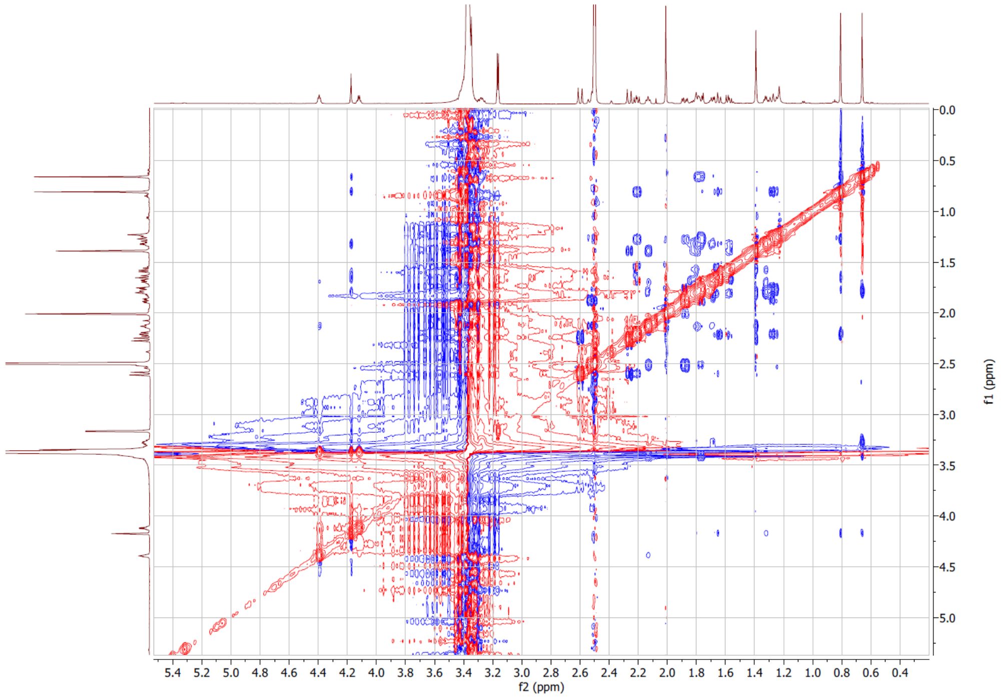

Figure S26. ROESY (DMSO-d_6) spectrum of compound **4**, at 25℃.

Mass Spectrum SmartFormula Report

Analysis Info

Analysis Name D:\Data\MS\data\202105\wangliyan_17A-2_pos_45_01_10725.d
Method LC_Direct Infusion_pos_70-500mz.m
Sample Name wangliyan_17A-2_pos
Comment

Acquisition Date 5/17/2021 11:24:11 AM

Operator SCSIO
Instrument maXis 255552.00029

Acquisition Parameter

Source Type	ESI	Ion Polarity	Positive	Set Nebulizer	0.4 Bar
Focus	Active	Set Capillary	4500 V	Set Dry Heater	180 °C
Scan Begin	70 m/z	Set End Plate Offset	-500 V	Set Dry Gas	4.0 l/min
Scan End	1500 m/z	Set Charging Voltage	0 V	Set Divert Valve	Waste
		Set Corona	0 nA	Set APCI Heater	0 °C

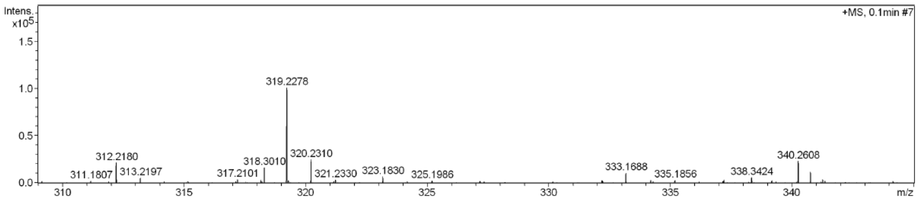

Meas. m/z	#	Ion Formula	Score	m/z	err [ppm]	err [mDa]	mSigma	rdb	e⁻ Conf	N-Rule
319.2278	1	C20H31O3	100.00	319.2268	3.1	1.0	14.6	5.5	even	ok
637.4473	1	C40H61O6	100.00	637.4463	-1.6	-1.0	51.2	10.5	even	ok

wangliyan_17A-2_pos_45_01_10725.d
Bruker Compass DataAnalysis 4.1 printed: 5/17/2021 11:40:03 AM by: SCSIO Page 1 of 1

Figure S27. HRESIMS spectrum of compound **4**.

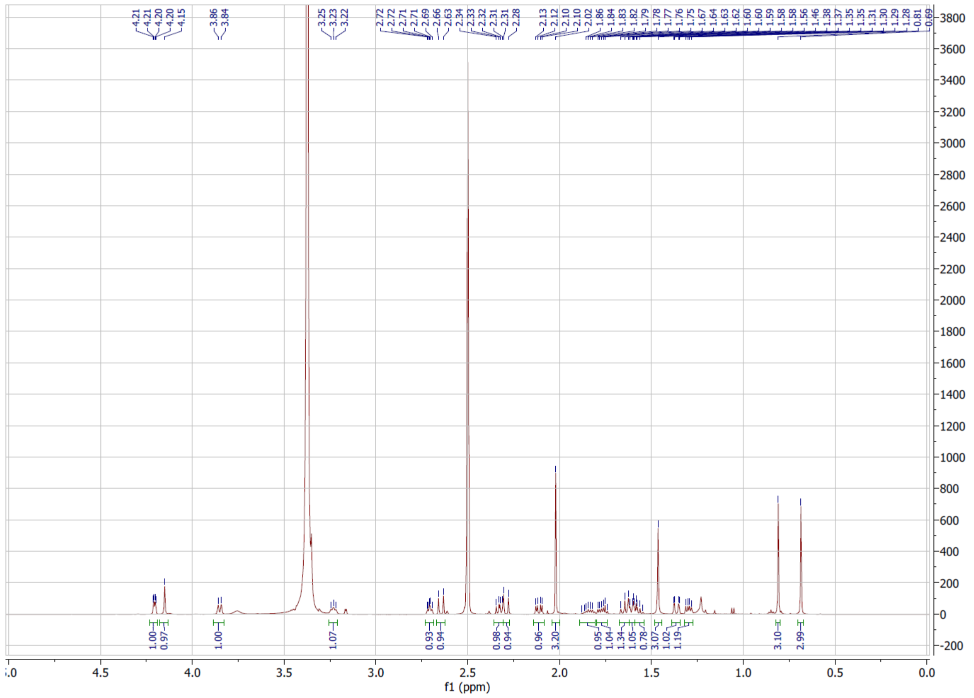

Figure S28. ^{1}H NMR (600 MHz, DMSO-d_6) spectrum of compound **5**, at 25°C.

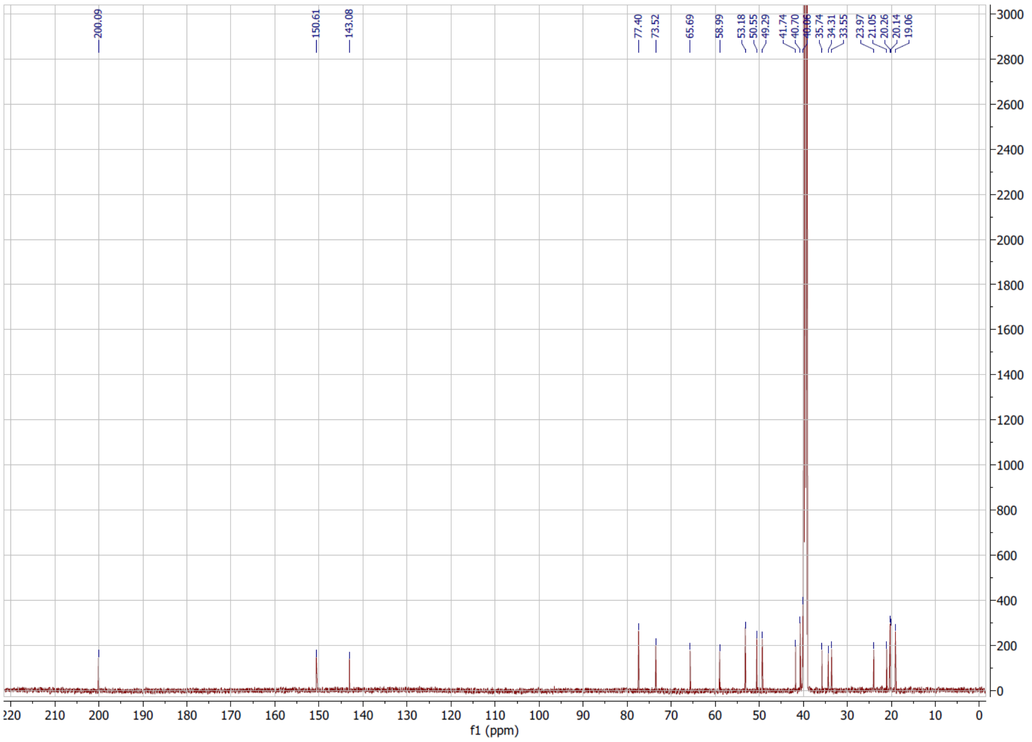

Figure S29. ^{13}C NMR (150 MHz, DMSO-d_6) spectrum of compound **5**, at 25°C.

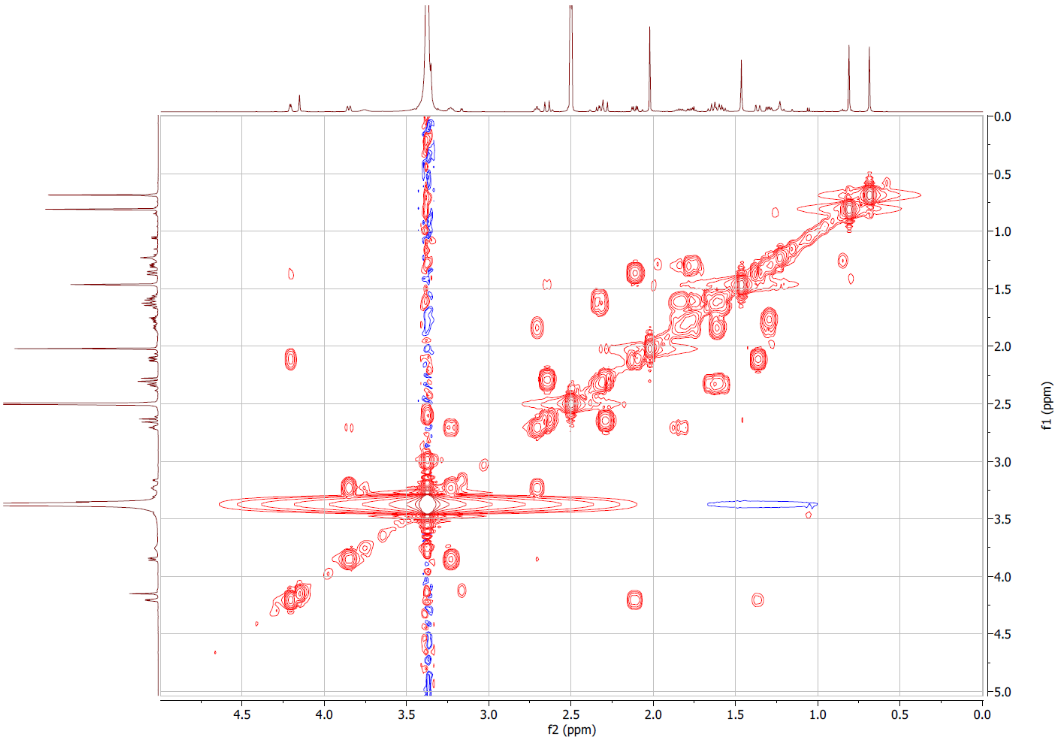

Figure S30. ^{1}H-^{1}H COSY (DMSO-d_6) spectrum of compound **5**, at 25°C.

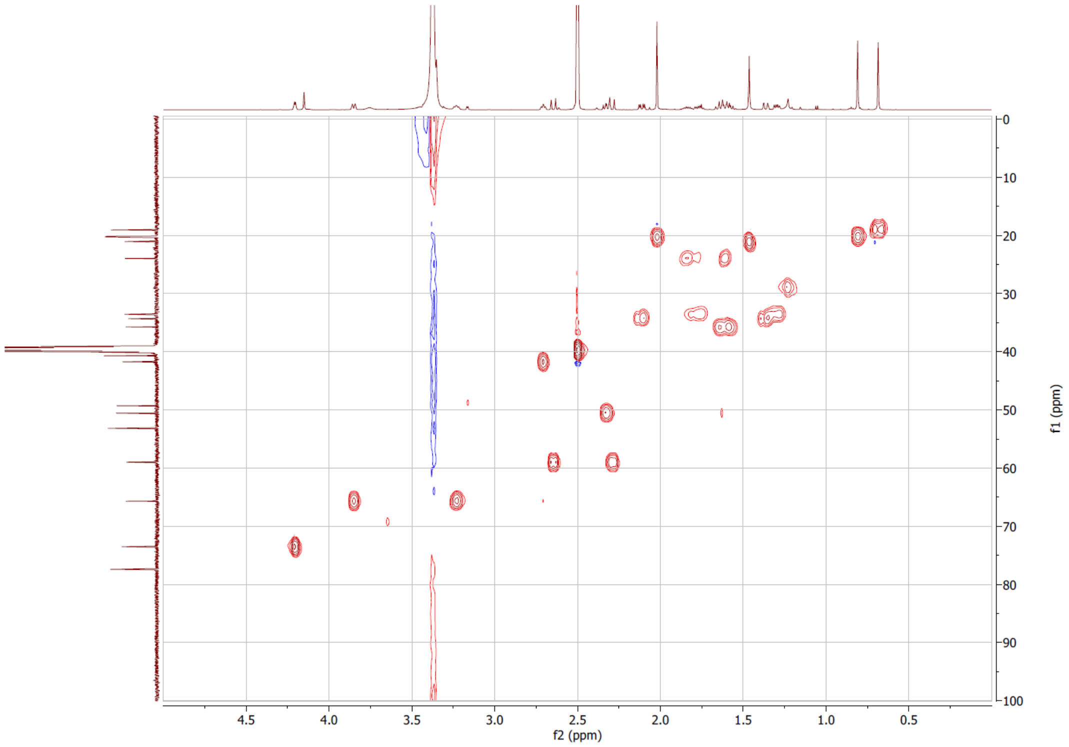

Figure S31. HSQC (DMSO-d_6) spectrum of compound **5**, at 25°C.

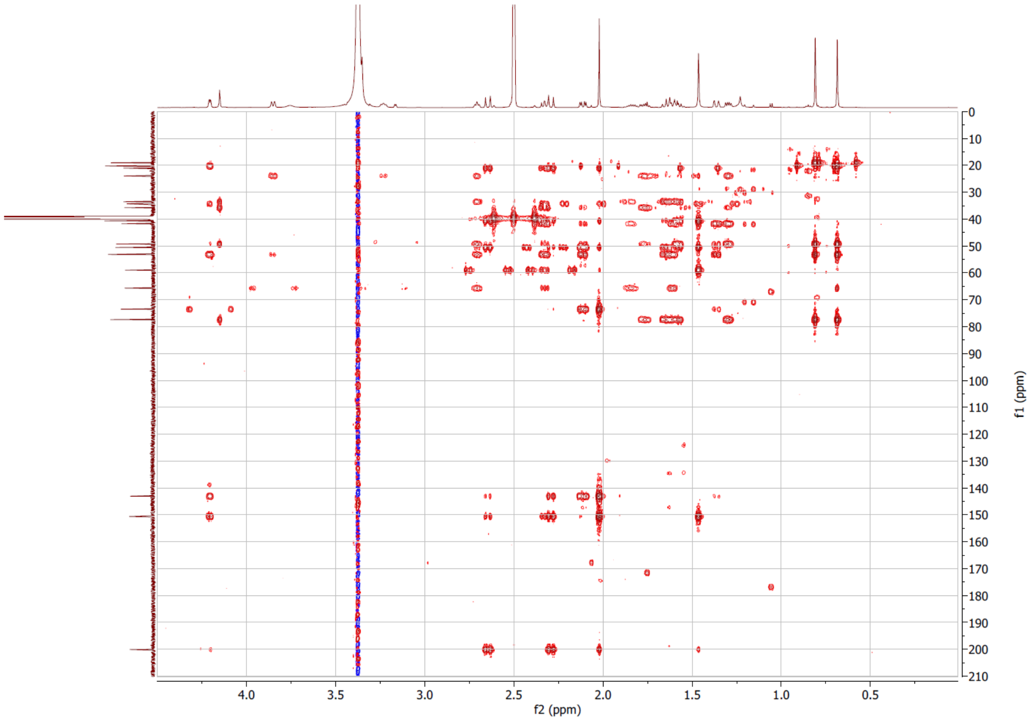

Figure S32. HMBC (DMSO-d_6) spectrum of compound **5**, at 25°C.

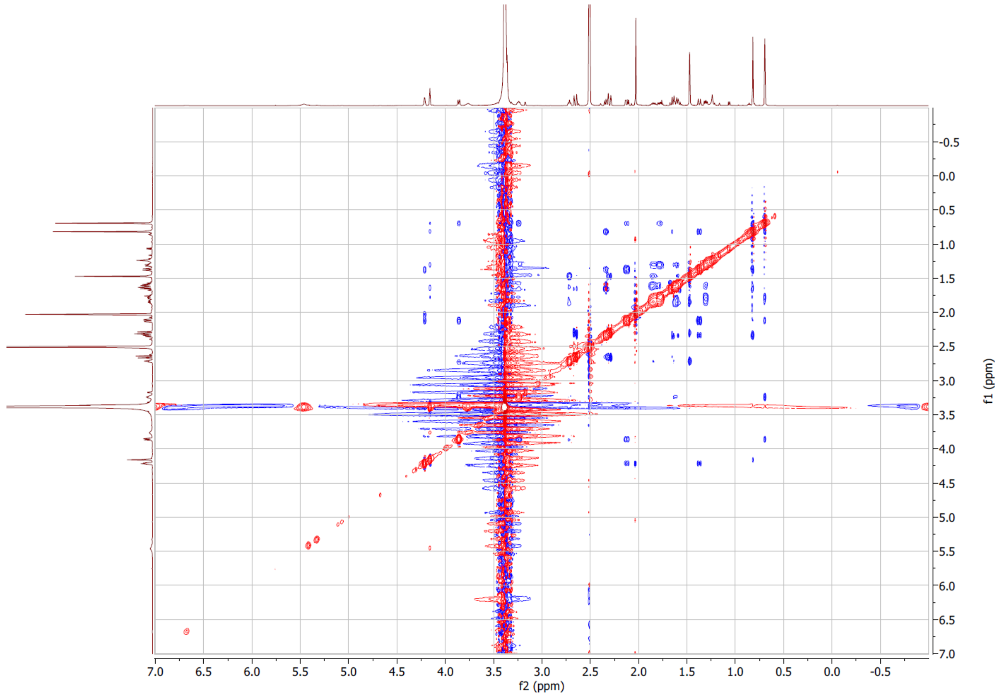

Figure S33. ROESY (DMSO-d_6) spectrum of compound **5**, at 25°C.

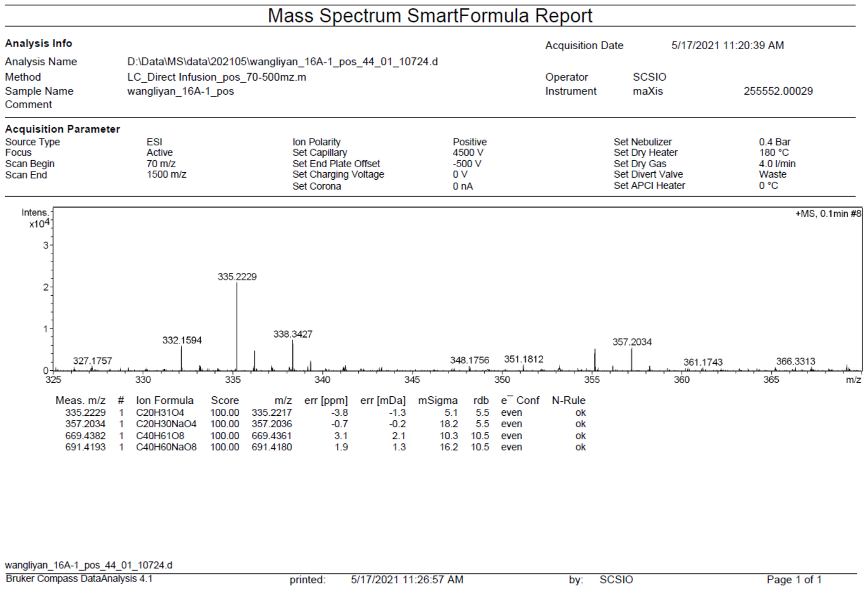

Mass Spectrum SmartFormula Report

**Analysis Info**		Acquisition Date	5/17/2021 11:20:39 AM
Analysis Name	D:\Data\MS\data\202105\wangliyan_16A-1_pos_44_01_10724.d		
Method	LC_Direct Infusion_pos_70-500mz.m	Operator	SCSIO
Sample Name	wangliyan_16A-1_pos	Instrument	maXis 255552.00029
Comment			

Acquisition Parameter

Source Type	ESI	Ion Polarity	Positive	Set Nebulizer	0.4 Bar
Focus	Active	Set Capillary	4500 V	Set Dry Heater	180 °C
Scan Begin	70 m/z	Set End Plate Offset	-500 V	Set Dry Gas	4.0 l/min
Scan End	1500 m/z	Set Charging Voltage	0 V	Set Divert Valve	Waste
		Set Corona	0 nA	Set APCI Heater	0 °C

Meas. m/z	#	Ion Formula	Score	m/z	err [ppm]	err [mDa]	mSigma	rdb	e⁻ Conf	N-Rule
335.2229	1	C20H31O4	100.00	335.2217	-3.8	-1.3	5.1	5.5	even	ok
357.2034	1	C20H30NaO4	100.00	357.2036	-0.7	-0.2	18.2	5.5	even	ok
669.4382	1	C40H61O8	100.00	669.4361	3.1	2.1	10.3	10.5	even	ok
691.4193	1	C40H60NaO8	100.00	691.4180	1.9	1.3	16.2	10.5	even	ok

wangliyan_16A-1_pos_44_01_10724.d
Bruker Compass DataAnalysis 4.1 printed: 5/17/2021 11:26:57 AM by: SCSIO Page 1 of 1

Figure S34. HRESIMS spectrum of compound **5**.

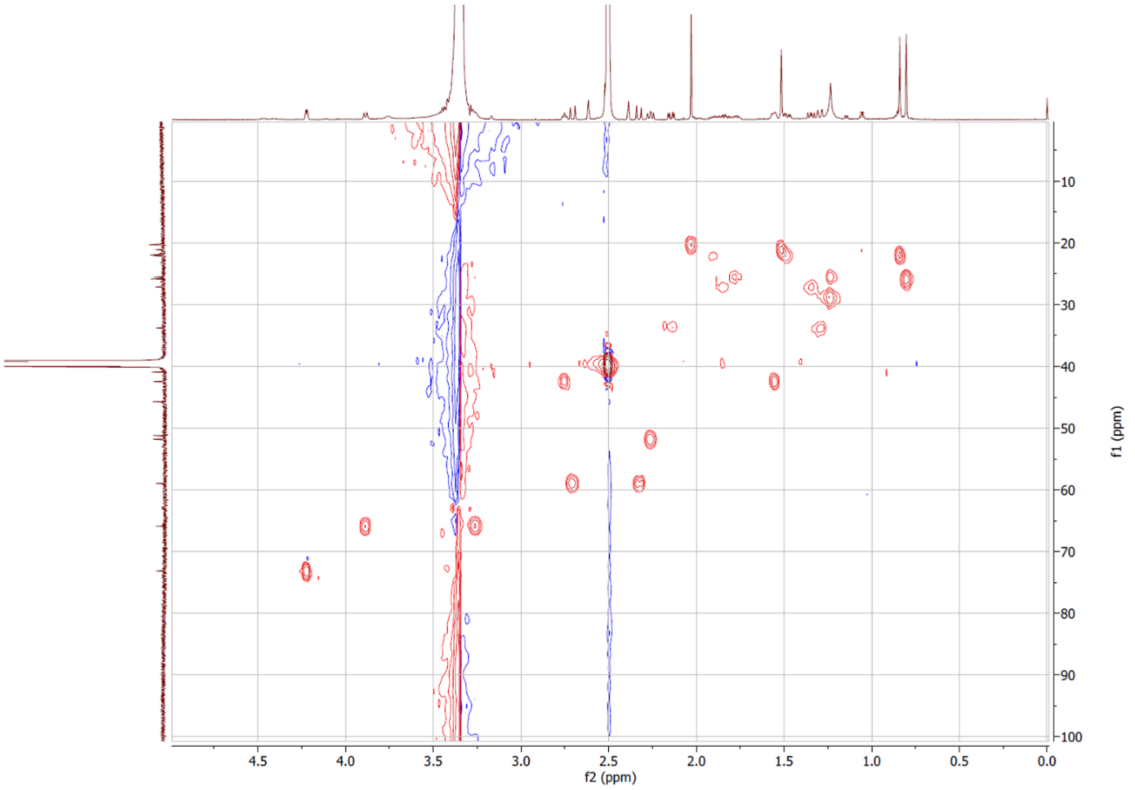

Figure S35. ^{1}H NMR (600 MHz, DMSO-d_6) spectrum of compound **6**, at 25°C.

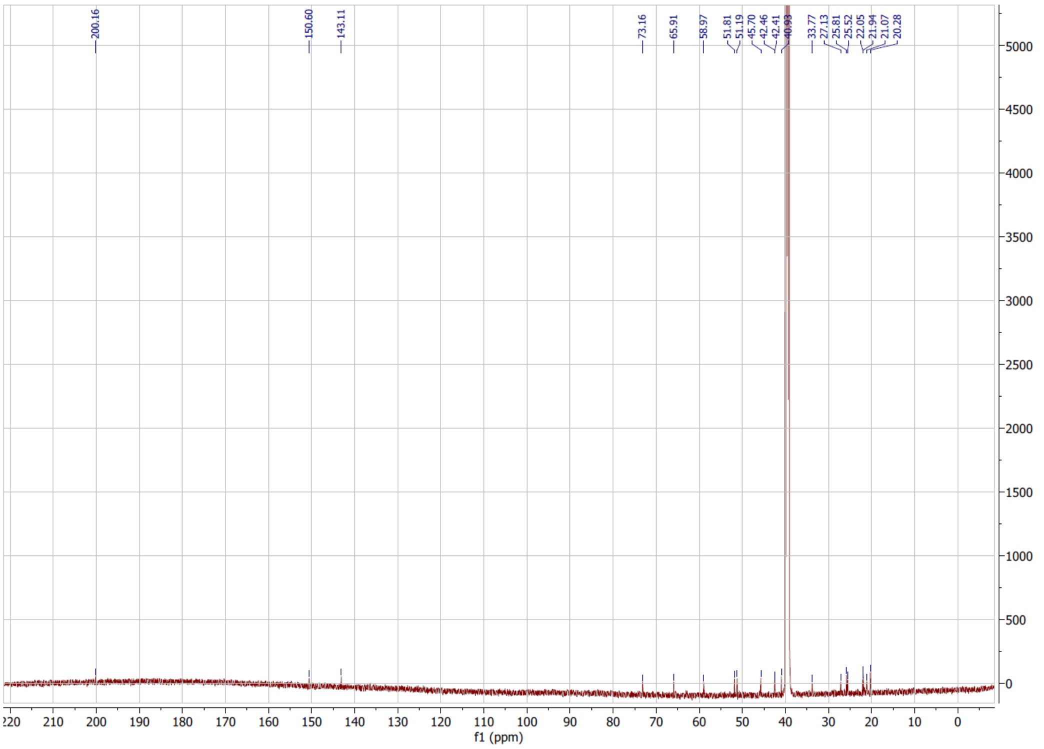

Figure S36. ^{13}C NMR (150 MHz, DMSO-d_6) spectrum of compound **6**, at 25°C.

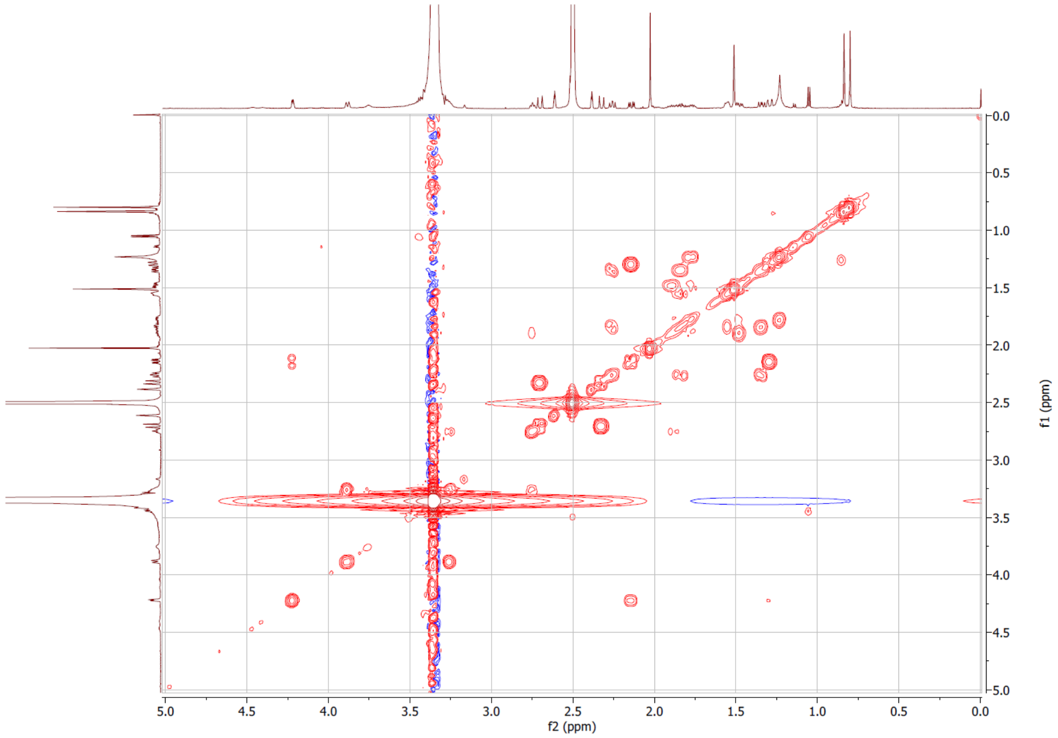

Figure S37. ^{1}H-^{1}H COSY (DMSO-d_6) spectrum of compound **6**, at 25°C.

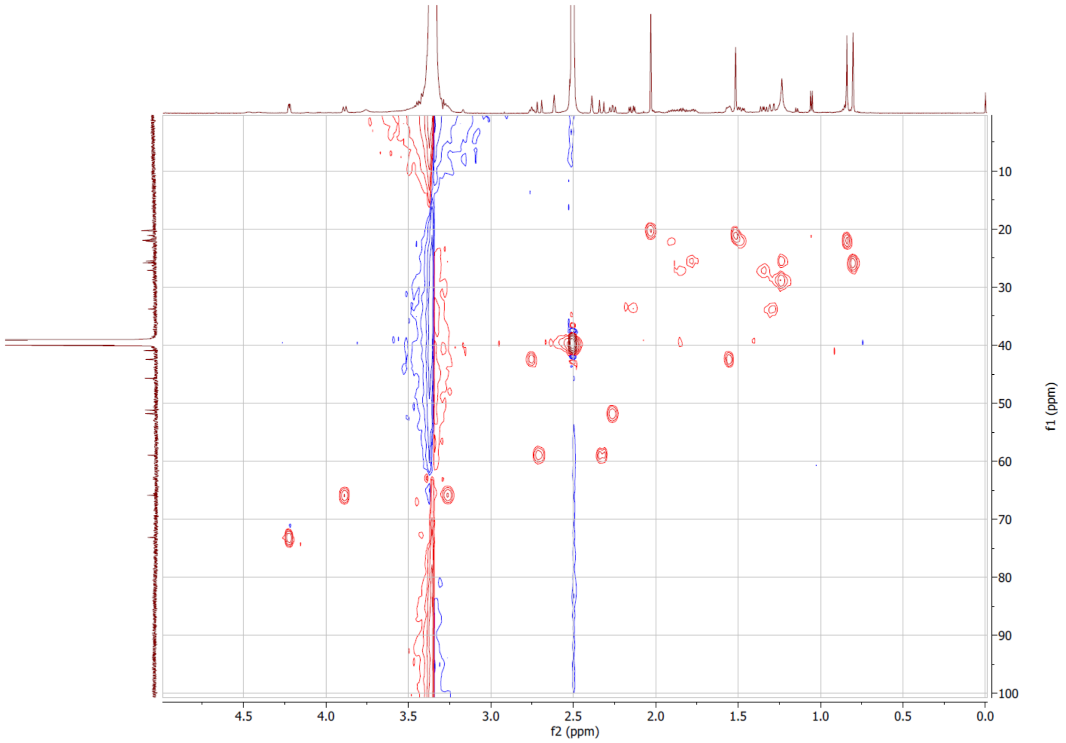

Figure S38. HSQC (DMSO-d_6) spectrum of compound **6**, at 25°C.

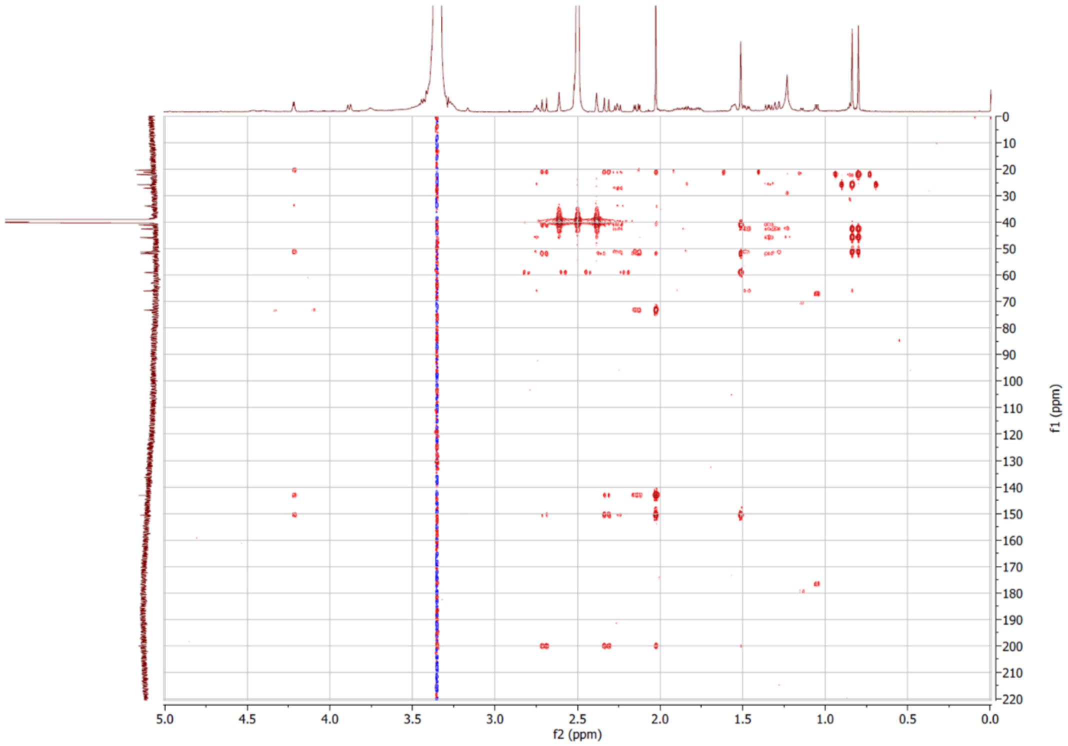

Figure S39. HMBC (DMSO-d_6) spectrum of compound **6**, at 25°C.

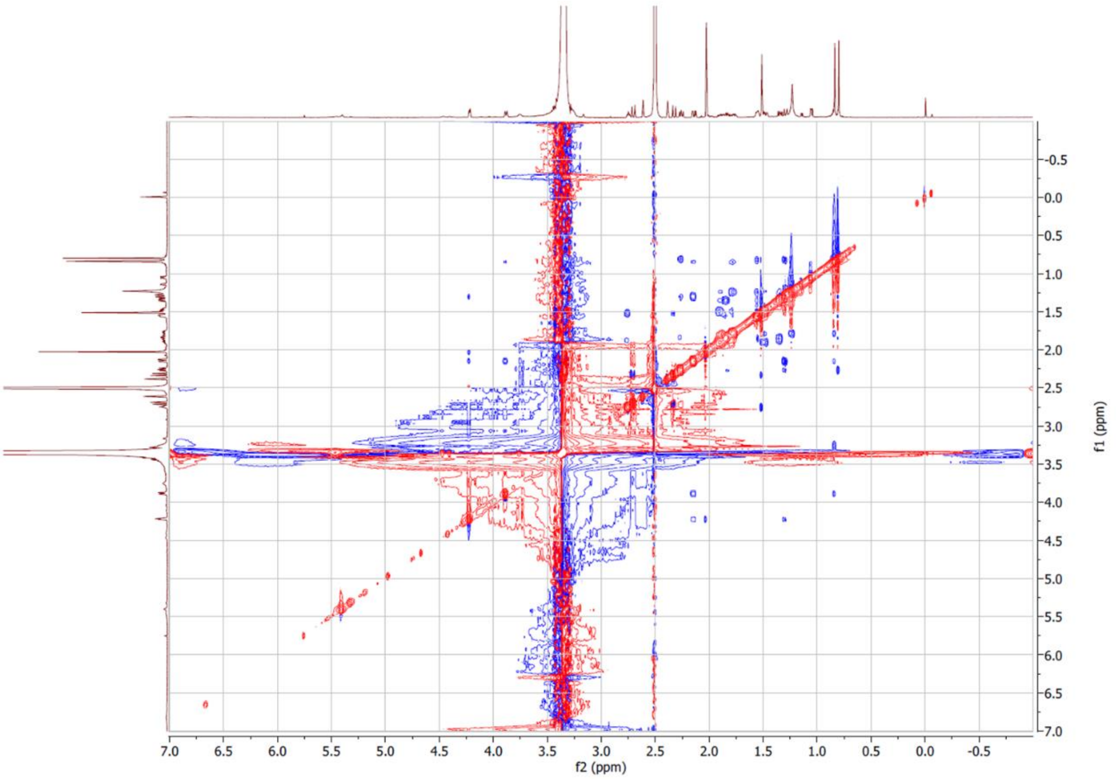

Figure S40. ROESY (DMSO-d_6) spectrum of compound **6**, at 25℃.

Mass Spectrum SmartFormula Report

Analysis Info

Analysis Name	D:\Data\MS\data\202105\wangliyan_8B-2_pos_43_01_10723.d	Acquisition Date	5/17/2021 11:17:09 AM
Method	LC_Direct Infusion_pos_70-500mz.m	Operator	SCSIO
Sample Name	wangliyan_8B-2_pos	Instrument	maXis 255552.00029
Comment			

Acquisition Parameter

Source Type	ESI	Ion Polarity	Positive	Set Nebulizer	0.4 Bar
Focus	Active	Set Capillary	4500 V	Set Dry Heater	180 °C
Scan Begin	70 m/z	Set End Plate Offset	-500 V	Set Dry Gas	4.0 l/min
Scan End	1500 m/z	Set Charging Voltage	0 V	Set Divert Valve	Waste
		Set Corona	0 nA	Set APCI Heater	0 °C

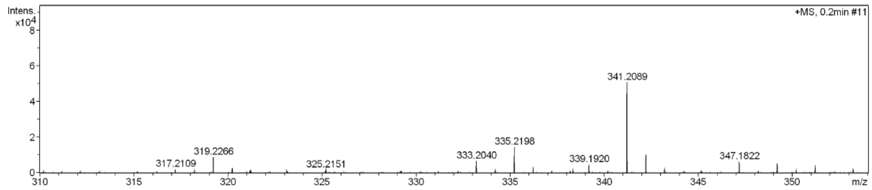

Meas. m/z	#	Ion Formula	Score	m/z	err [ppm]	err [mDa]	mSigma	rdb	e⁻ Conf	N-Rule
319.2266	1	C20H31O3	100.00	319.2268	0.6	0.2	66.1	5.5	even	ok
341.2089	1	C20H30NaO3	100.00	341.2087	-0.5	-0.2	12.5	5.5	even	ok
637.4468	1	C40H61O6	100.00	637.4463	-0.8	-0.5	37.0	10.5	even	ok
659.4297	1	C40H60NaO6	100.00	659.4282	2.2	1.5	52.4	10.5	even	ok

wangliyan_8B-2_pos_43_01_10723.d

Bruker Compass DataAnalysis 4.1 printed: 5/17/2021 11:24:11 AM by: SCSIO Page 1 of 1

Figure S41. HRESIMS spectrum of compound **6**.

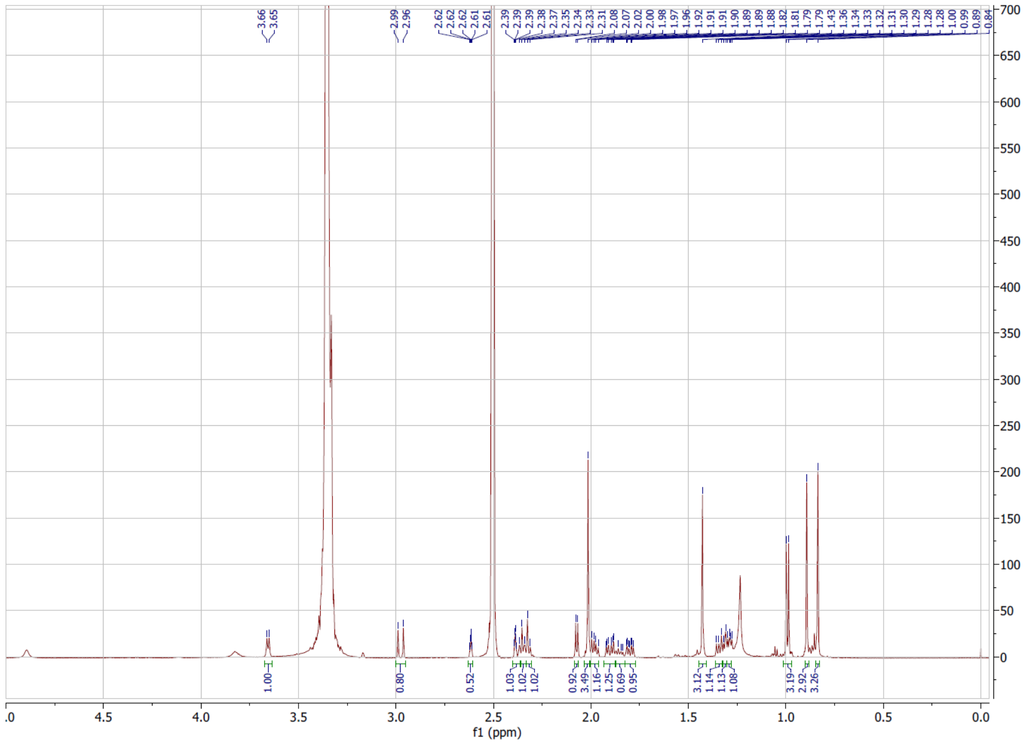

Figure S42. ^{1}H NMR (600 MHz, DMSO-d_6) spectrum of compound **7**, at 25°C.

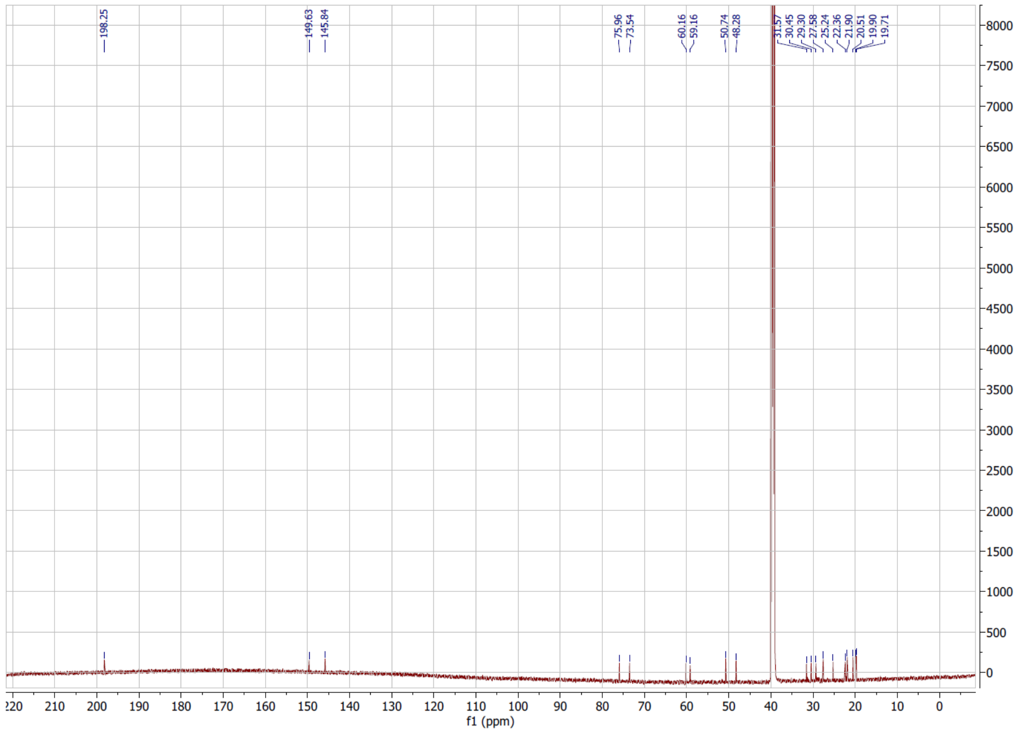

Figure S43. ^{13}C NMR (150 MHz, DMSO-d_6) spectrum of compound **7**, at 25°C.

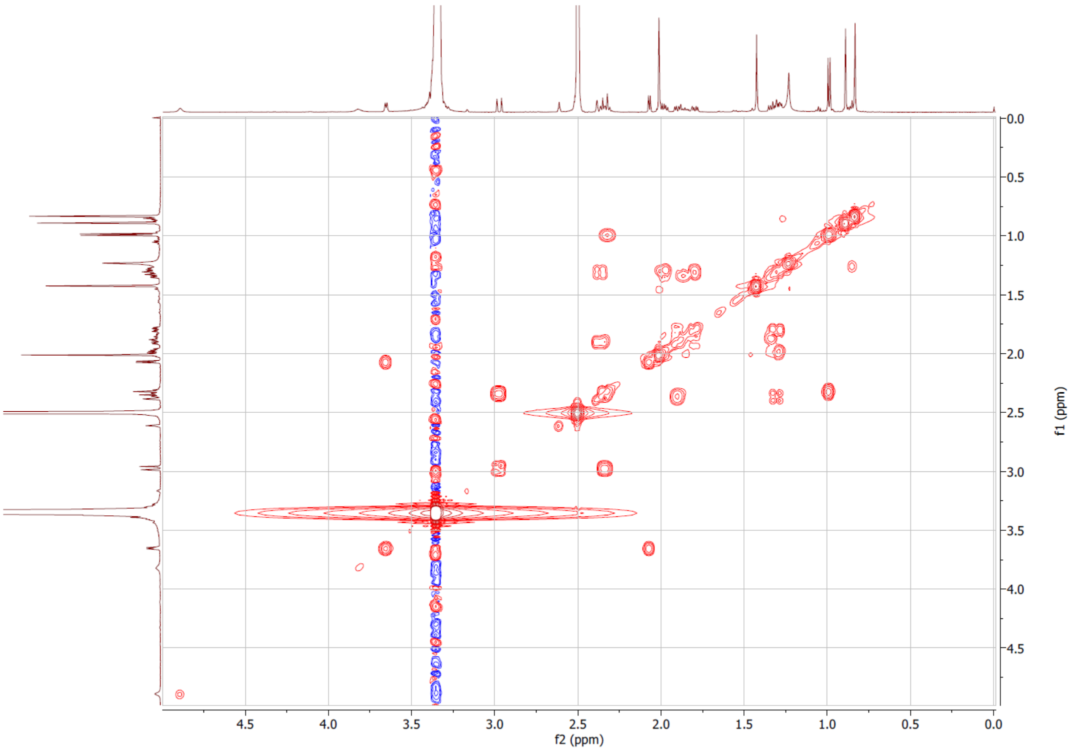

Figure S44. ^{1}H-^{1}H COSY (DMSO-d_6) spectrum of compound **7**, at 25°C.

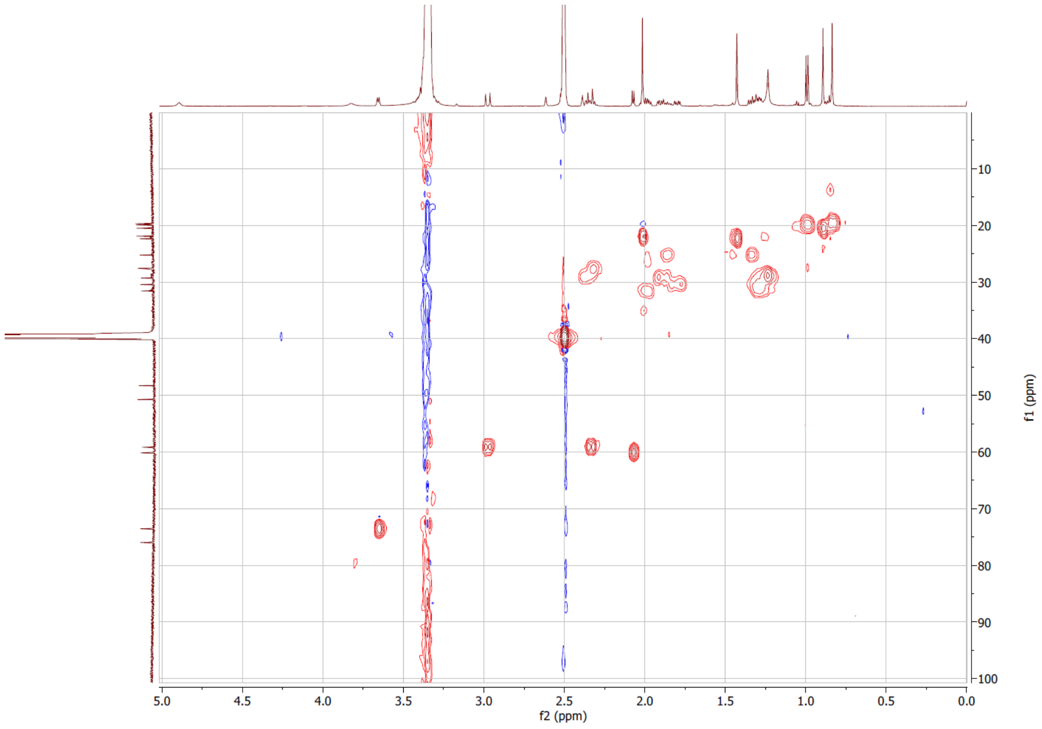

Figure S45. HSQC (DMSO-d_6) spectrum of compound **7**, at 25°C.

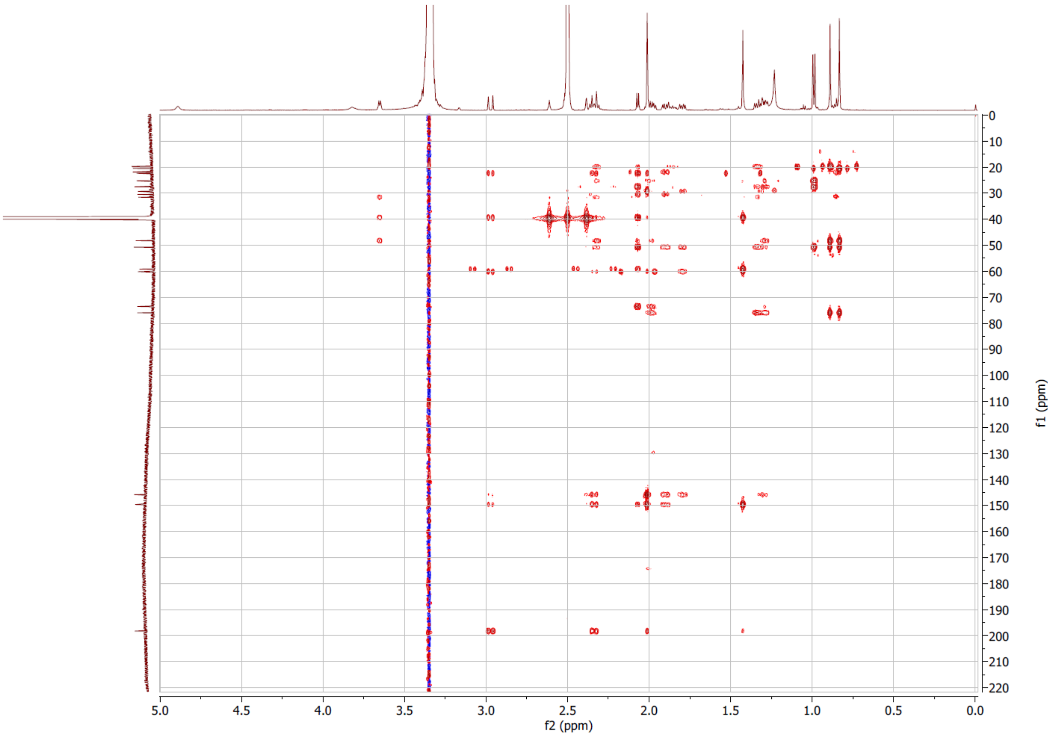

Figure S46. HMBC (DMSO-d_6) spectrum of compound **7**, at 25°C.

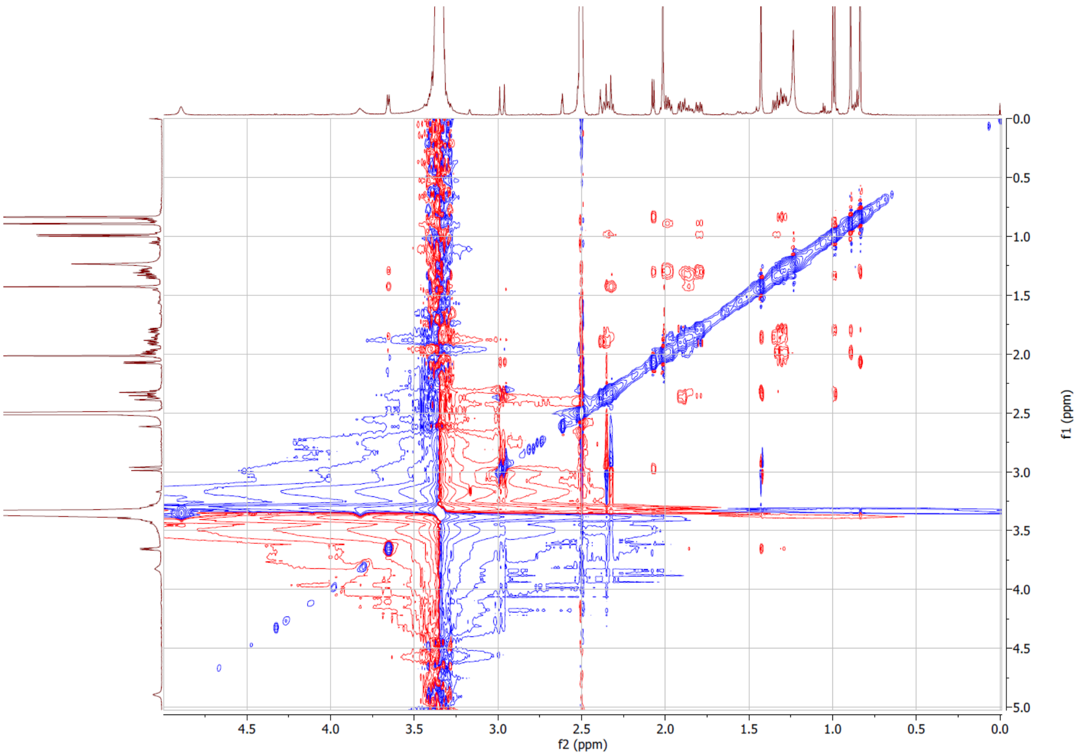

Figure S47. ROESY (DMSO-d_6) spectrum of compound **7**, at 25°C.

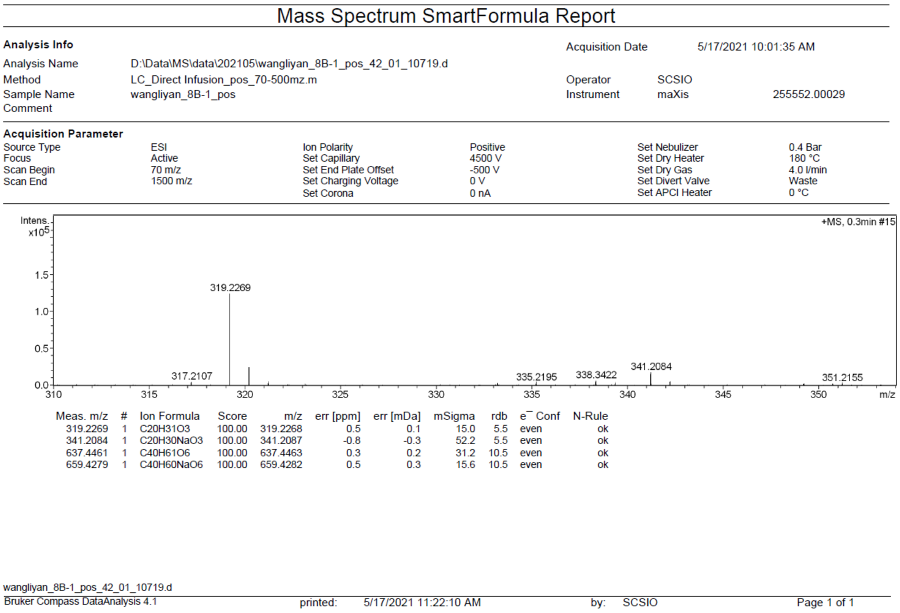

Mass Spectrum SmartFormula Report

Analysis Info

Analysis Name	D:\Data\MS\data\202105\wangliyan_8B-1_pos_42_01_10719.d	Acquisition Date	5/17/2021 10:01:35 AM	
Method	LC_Direct Infusion_pos_70-500mz.m	Operator	SCSIO	
Sample Name	wangliyan_8B-1_pos	Instrument	maXis	255552.00029
Comment				

Acquisition Parameter

Source Type	ESI	Ion Polarity	Positive	Set Nebulizer	0.4 Bar
Focus	Active	Set Capillary	4500 V	Set Dry Heater	180 °C
Scan Begin	70 m/z	Set End Plate Offset	-500 V	Set Dry Gas	4.0 l/min
Scan End	1500 m/z	Set Charging Voltage	0 V	Set Divert Valve	Waste
		Set Corona	0 nA	Set APCI Heater	0 °C

Meas. m/z	#	Ion Formula	Score	m/z	err [ppm]	err [mDa]	mSigma	rdb	e⁻ Conf	N-Rule
319.2269	1	$C_{20}H_{31}O_3$	100.00	319.2268	0.5	0.1	15.0	5.5	even	ok
341.2084	1	$C_{20}H_{30}NaO_3$	100.00	341.2087	-0.8	-0.3	52.2	5.5	even	ok
637.4461	1	$C_{40}H_{61}O_6$	100.00	637.4463	0.3	0.2	31.2	10.5	even	ok
659.4279	1	$C_{40}H_{60}NaO_6$	100.00	659.4282	0.5	0.3	15.6	10.5	even	ok

wangliyan_8B-1_pos_42_01_10719.d

Bruker Compass DataAnalysis 4.1 printed: 5/17/2021 11:22:10 AM by: SCSIO Page 1 of 1

Figure S48. HRESIMS spectrum of compound **7**.

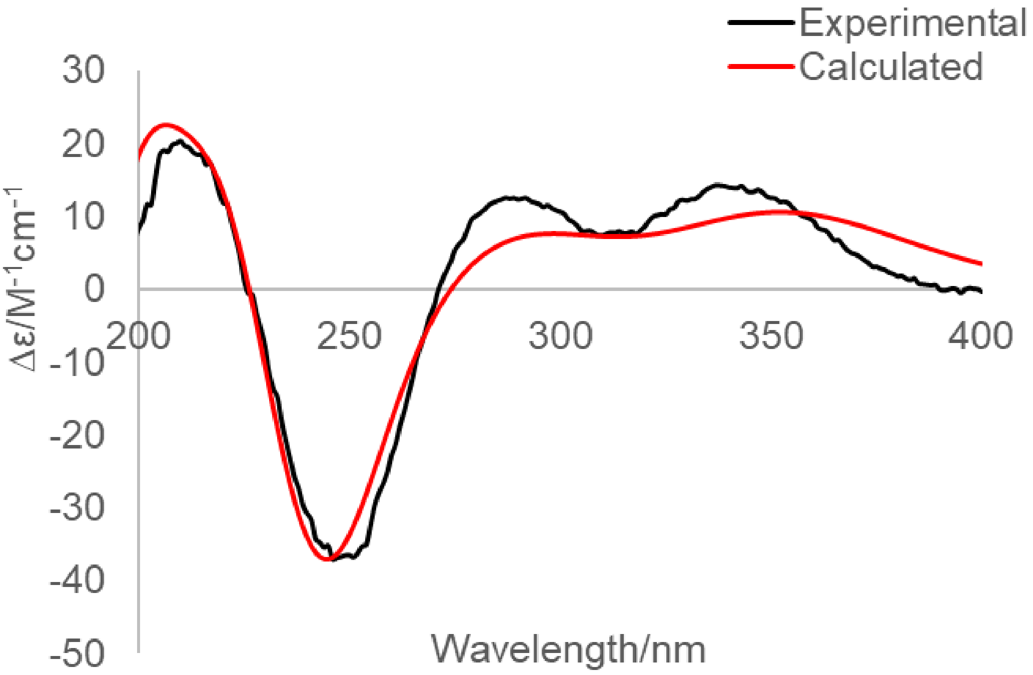

Figure S49. Comparison of the calculated ECD spectrum of (2*S*, 5*R*, 6*R*, 8*S*, 13*S*, 14*S*)-**1** at the B3LYP/6-311+G(d, p) level with the experimental ECD spectrum of **1** in MeOH, σ = 0.41 eV, shift = +10 nm.

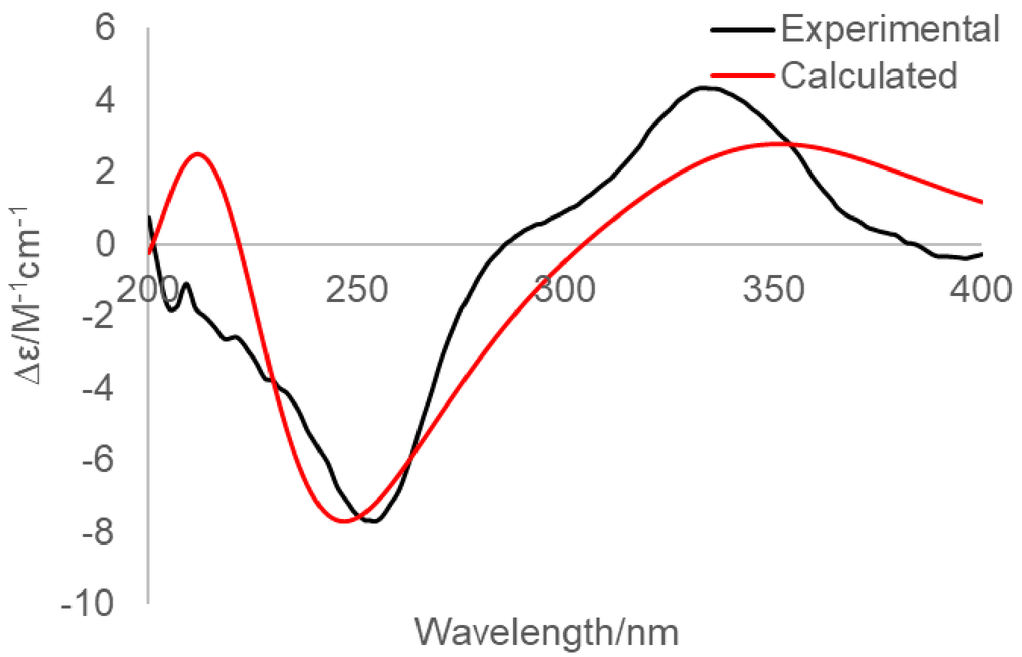

Figure S50. Comparison of the calculated ECD spectrum of 2*S*, 5*R*, 6*R*, 13*S*, 14*S*)-**4** at the B3LYP/6-311+G(d, p) level with the experimental ECD spectrum of **4** in MeOH, σ = 0.49 eV, shift = +10 nm.

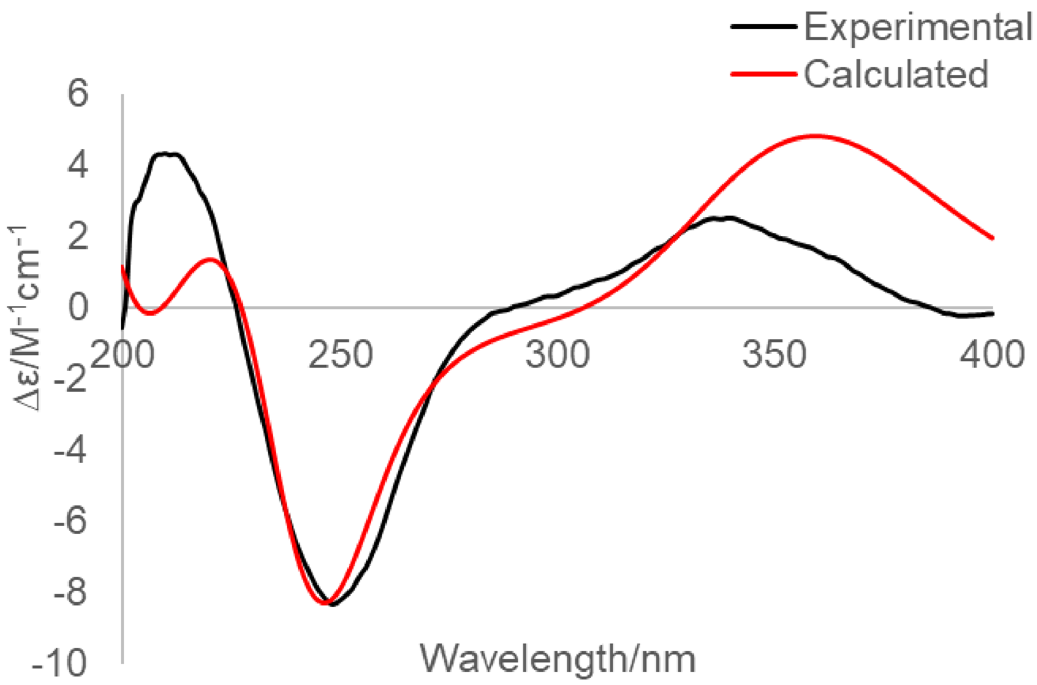

Figure S51. Comparison of the calculated ECD spectrum of (2*S*, 5*R*, 6*R*, 8*S*, 13*S*, 14*S*)-**5** at the B3LYP/6-311+G(d, p) level with the experimental ECD spectrum of **5** in MeOH, σ = 0.40 eV, shift = +15 nm.

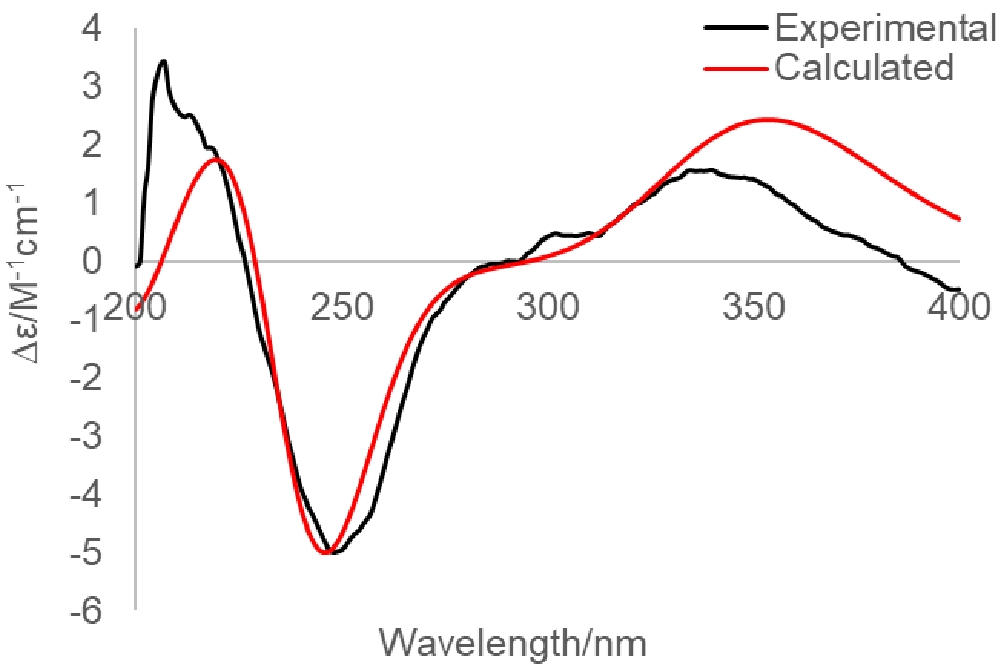

Figure S52. Comparison of the calculated ECD spectrum of (2*S*, 5*R*, 6*R*, 8*S*, 13*S*, 14*S*)-**6** at the B3LYP/6-311+G(d, p) level with the experimental ECD spectrum of **6** in MeOH, σ = 0.39 eV, shift = +9 nm.

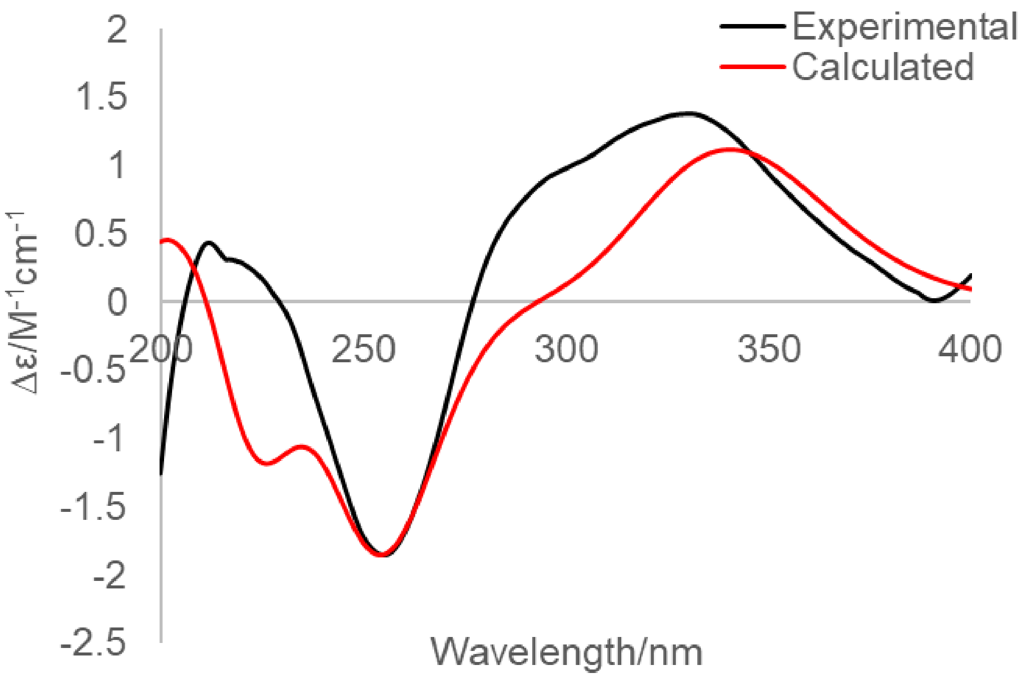

Figure S53. Comparison of the calculated ECD spectrum of (2*S*, 5*R*, 6*R*, 13*S*, 14*S*, 15*S*)-**7** at the B3LYP/6-311+G(d, p) level with the experimental ECD spectrum of **7** in MeOH, σ = 0.34 eV, shift = -2 nm.
